# Understanding Temporal Social Dynamics in Zoo Animal Management: An Elephant Case Study

**DOI:** 10.3390/ani10050882

**Published:** 2020-05-19

**Authors:** Ellen Williams, Samantha Bremner-Harrison, Carol Hall, Anne Carter

**Affiliations:** School of Animal, Rural and Environmental Sciences, Nottingham Trent University, Brackenhurst Campus, Southwell, Nottinghamshire NG25 0QF, UK; samantha.bremnerharrison@ntu.ac.uk (S.B.-H.); carol.hall@ntu.ac.uk (C.H.); anne.carter@ntu.ac.uk (A.C.)

**Keywords:** evidence-based management, social groups, social behaviour, social networks, temporal dynamics, welfare, zoo elephant

## Abstract

**Simple Summary:**

Zoo animal management can lead to disruption in social groups and poor individual welfare. Animals who display natural fluctuations in their wild social structure may be more difficult to cater for within zoos. The proactive management of animal social groups has potential implications for the positive welfare of individuals. Here, we used elephants as a case study to enhance the understanding of potential group dynamics in zoo animal social groups. Social interactions were defined as positive physical, positive non-physical, negative physical or negative non-physical. Data were collected over 12 months to investigate temporal dynamics in social networks. Positive social interaction networks were more interlinked than negative interaction networks. Social networks were fluid, but they did not follow a seasonal pattern. The results demonstrate the importance of understanding social networks and social behaviour over extended periods of time. Consideration of temporal changes in social relationships will enable and support evidence-based management. Such management will lead to the improved welfare of socially housed zoo species, through increased understanding and the recognition of the impact of management actions on welfare. In order to ensure the welfare of managed animals is not impinged by husbandry routines or breeding programmes, management must be led by knowledge of social relationships.

**Abstract:**

Zoo animal management procedures which lead to changes to social groups can cause disruption in social hierarchies and the temporary breakdown of social relationships. Animals have different roles in social networks. Understanding individual positions in social networks is important for effective management and ensuring positive welfare for all animals. Using elephants as a case study, the aim of this research was to investigate temporal social dynamics in zoo animals. Behavioural data were collected between January 2016 and February 2017 from 10 African and 22 Asian elephants housed at seven zoos and safari parks in the UK and Ireland. Social interactions were defined as positive physical, positive non-physical, negative physical or negative non-physical. Social network analysis explored social relationships including the fluidity of networks over time and dyadic reciprocity. Social interaction networks were found to be fluid but did not follow a seasonal pattern. Positive interaction networks tended to include the entire social group whereas negative interactions were restricted to specific individuals. Unbalanced ties were observed within dyads, suggesting potential inequalities in relationships. This could impact on individual experiences and welfare. This research highlights subtle temporal dynamics in zoo elephants with the potential for species-level differences. Similar temporal dynamics may also be present in other socially housed zoo species. This research thus provides evidence for the importance of understanding the social networks of zoo animals over longer periods of time. Understanding social networks enables pro-active and evidence-based management approaches. Further research should seek to identify the minimum sampling efforts for social networks in a range of species, to enable the implementation of regular monitoring of social networks and thus improve the welfare of social species under human care.

## 1. Introduction

Social groups and the opportunity to engage in social interactions and develop relationships can benefit animals in a number of ways. This includes cooperation to achieve common goals, enhancement of physical and psychological well-being and enhanced reproductive output [1]. Animal “friendships” or relationships can be assessed via proximity to others [2,3] or through physical interactions [2], with strong social relationships being characterised by frequent and symmetrical affiliative social interactions that are consistent over time [2]. Tactile behaviour is an important part of the maintenance of social relationships in several mammalian species [4], playing a role in establishing, maintaining and reinforcing social bonds [5,6].

Within zoos, the appropriate management of social groups is considered one of the most important, but difficult tasks to achieve. Species with fission-fusion social dynamics have the potential to be the most difficult to cater for within a zoo [7]. Stable social groups are positive, supportive influences on members [7], providing opportunities for interactions that promote positive welfare [8] and buffer stress [9]. Reduced group stability can lead to changes in social group hierarchies and relationships and have consequent negative effects on welfare. In primates, reduced group stability can lead to group fissions. However, within zoos, group fission may not be possible and that can lead to outbreaks of aggression or the breakdown of social hierarchies [10]. Likewise, social instability in managed domestic animals can also lead to reduced welfare. In horses (*Equus caballus*) housed in stable social groups, agonistic behaviour was significantly lower than those housed in unstable social groups [11], and in cattle (*Bos taurus*) regrouping has led to negative impacts on emotional and physiological wellbeing, health and resistance [12]. Furthermore, stable social groups in goats (*Capra aegagrus hircus*) has led to the development of affiliative relationships, a reduction in agonistic relationships and increased group cohesion [13].

Identification of key individuals within a social group can help to ensure management decisions, such as moving an individual for breeding, do not impact upon the overall stability of social groups in animals under human care [14]. However, in order to understand animal social relationships, it is first important to understand whether temporal changes are likely to be present within animal social groups. If social relationships are static it may be possible to make evidence-based management decisions based on a single observation point. If structures are more fluid then observations should be taken over an extended time period, in order to provide a more accurate picture. 

Elephants are an excellent species to use as a case study when conducting social behaviour and social group structure research. In situ, elephants engage in fission-fusion relationships [15,16,17,18]. Within zoos, elephants are held in relatively static social groups although they may be subject to social group changes as part of routine management [19]. Previous researchers have highlighted a plethora of welfare problems associated with inappropriate social housing in zoo elephants [20,21]. Wild African elephants (*Loxodonta africana*) show variability in social structures over time [22], which are associated with environmental factors. During the dry season social cohesion decreases, which is believed to be related to the capacity of the environment to support larger groups [15,23]. Similar social group structure changes in relation to resource availability are observed in other large herbivores, such as Przewalski’s horse (*Equus ferus przewalskii*) and Asiatic wild asses (*Equus hemionus*) [24]. Whereas in wild giraffe (*Giraffa camelopardalis*), social group structures are driven by kinship and social preferences [25]. Wild Asian elephants (*Elephas maximus*) also show variability in social structures, but this variability is believed to not be driven by environmental factors, rather they are considered to be more affected by social than ecological factors [17,26].

The strength of social relationships differs in wild Asian and African elephants. In Asian elephants, dyadic ties are generally weaker than those seen in African elephants. However, despite an overall weakness in social ties, the majority of individuals will exhibit a few strong and consistent relationships that are maintained over time [26]. Social interactions in African elephants are non-random [22]; networks of African elephants are far more interconnected than the Asian elephant networks; each individual is more closely connected to more individuals by fewer steps than in the Asian elephant network (that is, associates of a female African elephant are more likely to be associated with one another than associates of a female Asian elephant) [17]. In Asian elephants, long-term fidelity to companions is variable but stability at the population level is indicative of some long-term stable associations [17]; female Asian elephants do not engage in completely random interactions, rather they “shuffle” amongst a set of preferred companions with individual variation at the dyadic level. Despite these differences in wild-type social structures, within UK and Irish zoos elephant management guidelines do not provide species-specific social management guidelines [27].

An imbalance in relationships has important potential effects on welfare, especially in negative interaction networks. Understanding relationships and advancing knowledge at the level of group and dyadic interactions provides the opportunity to improve animal welfare on an individual level, by informing decisions relating to housing and husbandry regimes. The opportunity to understand social networks and identify roles of key individuals ensures management decisions can be made and executed with minimal effects on the overall stability of the social group [28]. Furthermore, regular sampling of social dynamics can allow managers the opportunity to detect changes to social relationships, allow early identification of potential areas of conflict, and put in place intervention strategies to prevent conflict escalation [29]. In elephants, dyadic relationships can identify changes such as a shift from a balanced relationship to an unbalanced relationship or an increase in negative social interactions towards one particular group member. Such subtle changes may be representative of hierarchical herd changes arising from the introduction of new elephants, births or deaths, puberty [30] or other unforeseen hormonal changes in older individuals.

The importance of evidence-based management in zoo animals has been increasingly recognised. Using elephants as a case study, the aim of this research was to investigate the stability of social relationships in zoos. Specifically, it was to identify whether individuals differ in their roles within social networks and to determine whether temporal dynamics are present in zoo social groups.

## 2. Materials and Methods

### 2.1. Ethics Statement

All research protocols were approved by the Nottingham Trent University School of Animal, Rural and Environmental Sciences School Ethics Group, Southwell, UK (reference number ARE188). Permission to conduct the study was granted by the participating zoos prior to commencement of data collection. Support for the study was obtained from the BIAZA Research Group, London, UK.

### 2.2. Subjects and Study Sites

Subjects were 10 African (1 male and 9 females) and 22 Asian (3 males and 19 females) elephants housed at seven zoos and safari parks in the UK and Ireland. Herd size ranged from two to nine. An additional individual housed at Zoo E was excluded from data analysis due to missing behavioural data (Table 1).

### 2.3. Data Collection

Data collection followed the methods detailed in Williams et al. [31]. For completeness, protocols are described in brief. Elephants were identified using visually discernible differences: height, size and shape of ears, length of tail and presence/absence of hair, scars and tattoos. Data were recorded via live and video observations. Live observations were conducted from public viewing areas during zoo visitor hours. Video footage was either provided by the study zoo from existing cameras (Zoo A, C and E), or cameras were temporarily installed on-site (Zoo D, F and G). See Williams et al. [31] for technical details of recording equipment.

The main data collection period ran from January 2016 to February 2017 (Table 2). Observations were undertaken by a single observer. Data were collected over a 5-day period each month with each 24 h day split into 12 × 2-h periods. Within each 2-h period data were randomly collected for 1 h. Observations were stopped whenever elephants were involved in keeper-initiated interactions (e.g., public feeding displays or training). There was a discrepancy in the hours of observations that were able to be undertaken across the study zoos due to external circumstances, e.g., failure of recording equipment, and it not always being possible to view all study elephants for the full duration of each observation period due to enclosure set-ups. Therefore, data were analysed as a proportion of total possible observations, to enable cross-zoo comparisons to be made.

### 2.4. Social Interactions

Scan sampling, at 30 s intervals, and instantaneous recording were employed to reduce sampling bias during the one-hour sampling period, e.g., only recording the first elephant to take part in an interaction, or to limit introducing an error in interpretation of the context of the interaction. Social interactions were split into positive and negative interactions. Interactions were considered to be positive if they were non-aggressive contact or non-aggressive approaches (e.g., touching with the trunk), and negative if they were instances of aggression or a reaction to aggressive behaviour (e.g., walking away from another elephant) [32,33]. Positive and negative social interactions were then further subdivided into physical and non-physical interactions (Table 3) [31]. Directionality of the interaction was recorded to establish reciprocity in dyadic relationships.

### 2.5. Data Analysis

Data analysis was split into two areas for analysis: (i) frequency of social interactions given by individual elephants and (ii) herd social matrices. Data were split into four equal quartiles, each comprising of one to two months of data collection (P1, P2, P3 and P4) (Table 3), to investigate the stability of herd dynamics over 12 months. Social behaviour was investigated in terms of frequency of interactions at the four data collection periods, and longitudinally between the first and last periods of data collection. Despite elephant species exhibiting different social structures in the wild, recommendations for zoo elephant management are not species-specific. Therefore, analysis was undertaken both in terms of separate elephant species (African and Asian) and also as a combined population to determine if species-level differences were present.

### 2.6. Frequency of Social Interactions Given by Individual Elephants

Statistical analyses were undertaken using SPSS Version 21 (SPSS Inc., Chicago, IL, USA). A Friedman’s test with a Wilcoxon post-hoc was undertaken to analyse whether the frequency of interactions given by individual elephants and within dyads changed at the different time points. Bonferroni adjustments were applied to post hoc analyses (reducing the significance value to *p* = 0.008) to cater for replicates in data analysis.

### 2.7. Fluidity in Herd Relationships Over Time and Dyadic Reciprocity

For the purposes of this research “herd relationships” are defined as the frequency of interactions between individual elephants within the whole herd. Two elephants were removed from the “fluidity in herd relationships” analysis due to missing data. E2 (Zoo A) passed away after the first period of data collection and E23 (Zoo E) was not born until after the second data collection period. Changes in herd relationships over time and reciprocity in dyads were assessed using Mantel tests undertaken in R (Version 1.1.383) using packages “ade4” and “vegan”. A total of 999 permutations were used per test, with the Pearson product moment correlation coefficient as the test statistic. Significance levels were set at 0.05. Tests of reciprocity were undertaken to determine whether dyadic social interactions were reciprocal (i.e., to determine whether the rate of interaction of elephant E1 directed towards E2 was correlated with the rate of interaction that E2 directed to E1). Mantel tests were undertaken to examine absolute reciprocity. No correlation between the matrix and its transpose indicated unidirectional interactions. The equality of relationships within the whole herd matrix was also assessed using simple ratio methods. Dyadic interactions were considered to be relatively balanced if the ratio of interactions given to interactions received was between 0.5:0.5 and 0.41:0.59.

Social interaction matrices were created for individual herds using the frequency of interaction data for positive physical, negative physical, positive non-physical and negative non-physical interactions. Matrices of mean interactions were created for each data collection period. Each period was then compared with the subsequent data collection period to examine changes in herd relationships over a longitudinal period. Therefore, the three initial analyses that were undertaken were (1) P1–P2, (2) P2–P3 and (3) P3–P4. In order to ascertain whether herd level social interactions differed between any of the time points further analyses were undertaken to compare the remaining time periods (4) P1–P3, (5) P1–P4 and (6) P2–P4.

### 2.8. Identification of Key Individuals in Social Networks

Social network analysis was used to represent relationships between individuals in the herds. Weighted diagraphs were constructed from each asymmetric matrix for each type of interaction (positive physical, negative physical, positive non-physical and negative non-physical) using UCINET 6.0 Version 1.00 (Harvard, MA, USA) [34] and NetDraw Version 2.160 (Lexington, KY, USA) [35]. Betweenness centrality was used to quantify the importance of individuals within the social groups [36]. The matriarch or elephant considered to be most senior in the social group was identified by the keepers at each collection.

## 3. Results

### 3.1. Change Over Time

When the data were analysed in terms of frequency of interactions given by each elephant, there were no significant differences for positive physical, negative physical and negative non-physical interactions between the four time periods (*p* > 0.05). Frequency of positive non-physical interactions was significantly different across the time periods (χ^2^(3) = 21.125, *p* < 0.001). Bonferroni-corrected post-hoc tests revealed differences between P1 and P3 (Z = −3.795, *p* < 0.008), P1 and P4 (Z = −2.822, *p* < 0.008) and P2 and P3 (Z = −2.865, *p* < 0.008) (Table 4).

When frequencies of interactions given by each elephant were split in terms of species there were no significant differences for positive physical, negative physical and negative non-physical interactions between the four time periods for Asian elephants (*p* > 0.05). The frequency of positive non-physical interactions was significantly different across the time periods (χ^2^(3) = 26.657, *p* < 0.001) (P1 (median, IQR): 3.96% (1.79–10.34); P2: 3.62% (0.76–7.84); P3: 0.93% (0.35–2.03); P4: 1.41% (0.85–2.72)). Bonferroni-corrected post-hoc tests revealed differences between P1 and P3 (Z = −3.597, *p* < 0.008), P1 and P4 (Z = −3.146, *p* < 0.008), P2 and P3 (Z = −3.389, *p* < 0.008) and P2 and P4 (Z = −2.659, *p* < 0.008). There were no significant differences for any of the studied interactions (positive physical, negative physical, positive non-physical and negative non-physical) between the four time periods for African elephants (*p* > 0.05).

When the frequency of social interactions given were analysed within dyads there were significant differences between the time periods for positive physical (χ^2^(3) = 11.912, *p* < 0.01) and positive non-physical (χ^2^(3) = 76.188, *p* < 0.001) interactions, and negative non-physical interactions (χ^2^(3) = 15.544, *p* < 0.01). There were no significant differences in the frequency of physical negative interactions across the study periods (*p* > 0.05). Median values at the time points for each interaction type are provided in Table 5. Bonferroni corrected post-hoc tests identified differences between P1 and P4 for positive physical interactions (Z = −3.198, *p* < 0.008). Positive non-physical interactions differed between P1 and the other three time periods (P2:Z = −5.531, *p* < 0.008; P3: Z = −7.951, *p* < 0.008; P4: Z = −5.086, *p* < 0.008), between P2 and P3 (Z = −4.755, *p* < 0.008) and between P3 and P4 (Z = −2.944, *p* < 0.008). Differences were recorded between P1 and the other three time periods for negative non-physical interactions (P2: Z = −3.157, *p* < 0.008; P3: Z = −3.029, *p* < 0.008; P4: Z = −4.037, *p* < 0.008) (Table 5).

When dyadic interactions were split in terms of species there were no significant differences for any of the studied interactions (positive physical, negative physical, positive non-physical and negative non-physical) between the four time periods for African elephants (*p* > 0.05). In Asian elephants, differences between the time periods were observed for positive physical (χ^2^(3) = 15.438, *p* < 0.001) (P1 (median, IQR): 0% (0–0.16); P2: 0% (0–0.73); P3: 0% (0–0.16); P4: 0.04% (0–0.86))., positive non-physical (χ^2^(3) = 82.501, *p* < 0.001) [P1 (median, IQR): 0.26% (0–1.12); P2: 0.10% (0–0.55); P3: 0.03% (0–0.21); P4: 0.13% (0.04–0.29)]. and negative non-physical interactions (χ^2^(3) = 13.790, *p* < 0.01) (P1 (median, IQR): 0% (0–0.06); P2: 0% (0–0.03); P3: 0% (0–0); P4: 0% (0–0)). There were no significant differences in the frequency of physical negat0ive interactions across the study periods (*p* > 0.05). Bonferroni corrected post-hoc tests identified differences between P1 and P4 for positive physical interactions (Z = −3.402, *p* < 0.008). Positive non-physical interactions differed between P1 and the other three time periods (P2: Z = −5.280, *p* < 0.008; P3: Z = −7.899, *p* < 0.008; P4: Z = −5.011, *p* < 0.008), between P2 and P3 (Z = −5.365, *p* < 0.008) and between P3 and P4 (Z = −3.789, *p* < 0.008). Differences were recorded between P1 and the other three time periods for negative non-physical interactions (P2: Z = −2.984, *p* < 0.008; P3: Z = −3.321, *p* < 0.008; P4: Z = −3.665, *p* < 0.008).

### 3.2. Reciprocity in Dyads

Herd interactions were considered balanced if mantel tests revealed a significant correlation between the matrix of social interactions and the inverse matrix. A summary of mantel test correlation scores for each study zoo is provided in Table 6. The most balanced network across all study zoos was the positive physical interaction network. Negative physical interaction networks were not balanced at any of the study zoos.

### 3.3. Herd Social Matrices and Identification of Key Individuals

Findings are presented on a zoo by zoo basis in Table 7. Significant values represent correlations between the matrices, which means that the frequency of interactions within dyads remained consistent for the entire herd at the compared data collection points. Non-significant values (NS) mean that dyadic interactions (of the whole herd) differed over time. Stability of the social network differed across the study zoos (Table 7). The stability of the negative physical interaction network could not be analysed at Zoos D, E and F due to an absence of negative physical interactions.

Sociograms were created to visually represent social relationships within the elephant herds (Figure 1 and Figure 2). More detailed sociograms are provided in Figures S1–S13. Betweenness centrality scores for each individual elephant are presented in Table 8. A higher value indicates a greater influence within the network.

**Figure 1:**
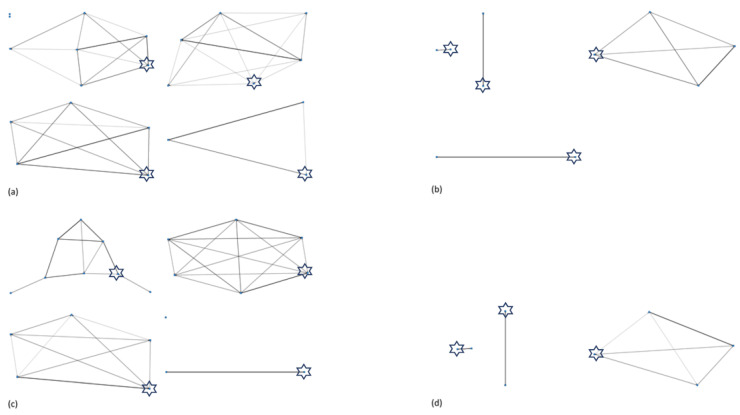
Sociograms depicting negative interaction networks in the study elephants. (**a**) Asian elephant negative non-physical interaction network, (**b**) African elephant negative non-physical interaction network, (**c**) Asian elephant negative physical interaction network, and (**d**) African elephant negative physical interaction network. Matriarch or elephant considered to be the most dominant herd member is highlighted with a star.

**Figure 2:**
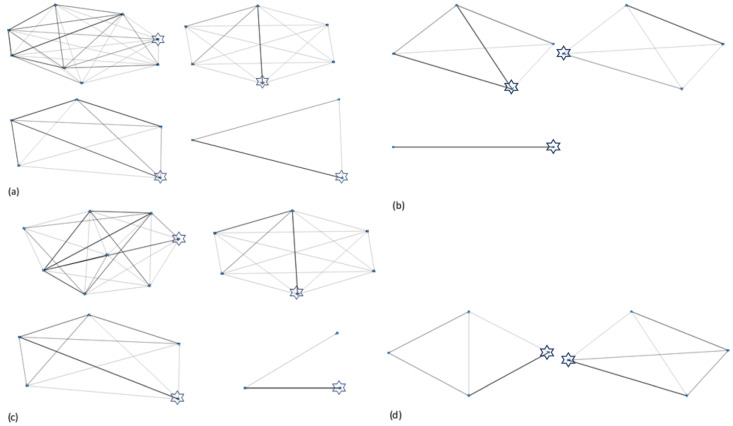
Sociograms depicting positive interaction networks in the study elephants. (**a**) Asian elephant positive non-physical interaction network, (**b**) African elephant positive non-physical interaction network, (**c**) Asian elephant positive physical interaction network, and (**d**) African elephant positive physical interaction network. Matriarch or elephant considered to be the most dominant herd member is highlighted with a star.

Positive interaction networks were more complex and interlinked than negative interaction networks with herds containing calves having the most complicated and interconnected networks (Figure 1 and Figure 2, Figures S4, S8 and S12). For the majority of the study zoos, the highest frequency of positive physical interactions was given or received by the matriarch, or elephant considered to be the most dominant in the group. The only exception to this was at zoo E where the greatest frequency of interactions was observed between a male and female calf (half-siblings). Not all elephants engaged in negative interaction networks (both physical and non-physical interactions), and interactions were generally low. Equal betweenness scores in the positive interaction networks indicate more equal relationships and less “key” individuals in the network. Balanced relationships were seen in both related and unrelated dyads (Table 9).

## 4. Discussion

Evidence-based social management, based on species knowledge, is extremely important for zoo and domestic animal welfare. Problems arising from unstable or inappropriate social groups have been highlighted in a number of exotic and domestic species including rhesus macaques (*Macaca mulatta*) [37,38], golden lion tamarins (*Leontopithecus rosalia*) [39], bottle nosed dolphins (*Tursiops aduncus*) [40], goats [13], cows [12] and horses [11]. Furthermore, the ability to cater for animal social needs within zoos has been identified as dependent on the species [7]. Research into social networks in zoo animals is increasing [36,37], but knowledge is still limited, especially for species with fission-fusion social dynamics. Close social associations in animals are beneficial, and having “friendships” is thought to enhance physical and physiological well-being [1]. Furthermore, a choice of conspecific partners has been highlighted as important for group compatibility and individual welfare [41,42,43]. This research takes elephants as a case study to investigate social interactions and determine the extent to which species with fission-fusion social dynamics exhibit temporal social dynamics within zoos.

In zoo elephants, the opportunity for appropriate social contact is considered more important for welfare than environmental space; social and management factors were important for more indicators of welfare (including performance of stereotypies, recumbence and prolactin production) than environmental space alone [19]. In this study, social networks showed varying levels in the consistency of interactions over time. Positive interaction networks were more complex and interlinked than negative interaction networks, and no extreme aggression was observed. Positive interactions strengthen social bonds of animals [1,4] and thus it would be expected that all individuals would benefit from engaging in these types of interactions to some degree. Positive interactions included the entire herd, whereas negative interactions were restricted to specific individuals or a subset of individuals from the entire social group. Variations were seen at the whole herd level and at the level of individual dyads.

### 4.1. Dyadic Relationships and Herd Hierarchies

Within zoos, animals may have altered social systems relative to their wild counterparts. Understanding dyadic relationships between zoo animals is an important consideration in zoo management as it can have implications for individual welfare [3], yet it is a vastly understudied area [2]. Harvey et al. [44] reported unbalanced social ties in the positive network in two groups of Asian elephants. Our findings contradict that; the positive physical network was the most equally reciprocated network, with the negative physical network showing no reciprocity in terms of whole herd interactions. Balanced relationships were seen in both related and unrelated dyads. These differences may have arisen because of the different periods of time over which the present study and the work by Harvey et al. [44] were undertaken, and the inclusion of a greater number of elephants in the current study.

Dyadic relationships may be due to relative hierarchical positions of individuals. Indeed, the highest frequency of positive physical interactions was given/received by matriarchs. Beyond the identification of a matriarch or elephant considered to be most senior in the social group, information on relative hierarchical positions of elephants within the study herds was not gathered due to keeper-identified, context-specific fluctuations in dominance hierarchies (McKenzie, personal communication). In the wild, African elephants form well-differentiated hierarchical relationships which are ordered by size and/or age [45] whereas Asian elephants have more sparse networks with age irregularities; opportunities for social segregation reduce the need for hierarchies [46]. Dominance hierarchies are expected to form and persist in response to socioecological pressures and competition over resources [46] and so provision of resources in a manner that prevents one individual monopolising a resource or active management to minimise competition [27] may reduce the need for a strict hierarchical herd structure within UK and Irish zoos.

### 4.2. Temporal Changes in Social Interactions

Social animals may show temporal dynamics. In order to gain a full understanding of social networks data must be collected in such a way that enables this to be investigated [47]. Within this study data were collected at four discrete time points over the period of 12 months, to enable this investigation to be undertaken. It is widely recognised that a number of species exhibit fission-fusion dynamics in the wild (e.g., Grevy’s zebra (*Equus grevyi*), giraffe, chimpanzees (*Pan troglodytes*), spider monkeys (*Ateles* sp.) and bottlenose dolphins), but factors influencing group changes are not always clear and the level of understanding is variable [25,48]. Research in giraffe suggests that fluidity in social dynamics is driven by kinship but also social preferences [25] whilst in Grevy’s zebra individual reproductive state influences association decisions [49]. In bottlenose dolphins, social structures are believed to be influenced by habitat and food availability and are brought about by a balance of the benefits and costs of forming associations [48]. In elephants, social group structural changes are believed to be driven predominantly by seasonality and associated ecological factors [17,22,50].

If temporal dynamics in social groups is resource-driven then variation in zoo populations would not be expected, due to consistency in resource provision throughout the year. However, if group dynamics are driven by social preferences then this may transfer over into zoos, and this would have potential implications for animal management. There is some disparity in recent work on elephant social behaviour, with some authors suggesting that Asian elephants in zoos show consistency in sociality over time [44], whereas others suggest that dyadic interactions, particularly tactile contact, can be variable [3]. In this study, consistency in temporal social dynamics was variable but elephants showed fluidity in their social relationships within zoos. The frequency of positive non-physical interactions given by each elephant differed across the data periods. Within dyads, frequency of positive physical, positive non-physical and negative non-physical interactions changed over time.

In this research, species differences were seen in social networks; African elephants showed no temporal dynamics in dyadic relationships and only one group showed temporal changes in positive physical and non-physical interactions at a herd level. Temporal changes observed in Asian elephant dyadic relationships suggests greater fluidity in these relationships than in African elephants, a factor which should be considered in zoo elephant management. Previously published research in the same groups of elephants indicated species-level differences in frequencies of positive non-physical social interactions, but not for positive physical, negative physical or negative non-physical interactions. Female Asian elephants show temporal stability in their close associative partners [51] as do female African elephants [22] and African bull elephants [52]. However, in both wild African and Asian elephants, temporal instability is observed in group size [15,16,17,18].

The lack of consistency in temporal dynamics indicates that social behaviour change is unlikely to be linked to seasonality, despite changes to management routines during winter months at some of the zoos. Seasonal changes in management routines, principally an increased amount of time in indoor enclosures, can have detrimental effects on elephant behaviour [21,53]. Three of the seven study zoos gave their elephants 24-h access to outside enclosures during the study months so this may have negated the effect of winter housing in terms of restriction to indoor environments. However, there are likely other changes such as changes to zoo opening hours, reduced keeper presence or reduced access to grazing, that could be impacting on behaviour, and so detailed management changes across a number of institutions should be considered in future work.

It is possible that the species-level differences observed were not indicative of biologically relevant species differences. There were more Asian elephants than African elephants in this study population and Asian elephant herds included on average larger, related herds with more calves/young elephants. Social behavioural changes may be observed as animals age [54]. In elephants, the social development of young individuals within herds, especially bull calves may impact on herd dynamics and relationships may change as animals age [55], and so temporal changes in social dynamics may be more expected when younger animals are present. Differences may also represent individual relationships or zoo-level effects on social relationships. Further studies involving multigenerational African elephant herds are recommended to quantify whether the differences observed were a genuine species-level difference. If there are species-level differences in social dynamics in zoo-housed African and Asian elephants then this lends support to the recommendation that species-level management guidelines should be implemented in relation to elephant social management.

It is important to note in research such as this that a lack of consistency in social interactions over time is not necessarily indicative of an incompatible social group or a cause for concern. Moreover, it highlights the recognition that zoo animals may exhibit fluidity in social dynamics, which is important to consider and understand, especially when moving individuals from social groups. The identification of preferred social partners at one point in time may not be a long-term preference and thus management decisions must not be made from a snapshot in time. The fluidity of interactions leads to a necessity to monitor for change. The presence of temporal changes in elephant social dynamics is important to consider in zoo elephant management, however, it is also extremely important to recognise that the presence of this behaviour in elephants may also be indicative of similar behavioural flexibility in other zoo species. These findings should thus be applied to the management of other social species, especially those who exhibit fission-fusion dynamics in wild populations.

### 4.3. Recommendations and Areas for Future Research

There were a number of factors that could have affected social relationships but were beyond the scope of the study to formally assess. For example, hormonal cycles or events occurring during the periods of data collection such as unique stimuli, maintenance work, fluctuations in visitor numbers or a change of keeping team. These would potentially have been present at all of the study zoos. The 12-month period of data collection should have minimised any effects. Future work in both elephants and other species should look to include consideration of the effect of management practices (e.g., winter or summer housing) and associated altered routines on social dynamics, to ensure animals are experiencing positive welfare year-round.

Elephant keepers have highlighted the need to provide elephants with appropriate social environments [43], yet the discrepancy in findings from the studies, which have since been undertaken, highlight the need for more rigorous research using identical methodologies to enable comparison and to aid understanding of zoo-elephant social relationships. An elephant’s welfare is affected by the social, physical and cognitive environment, all of which are moving to a greater or lesser extent [30]. The results of this research highlight the potential for subtle temporal changes in zoo elephant social relationships. Furthermore, they highlight the potential for species-level differences. It is thus advocated that social behaviour in African and Asian elephants housed in zoos be researched more fully, in a greater range of facilities, to enable incorporation of this knowledge into elephant management guidelines if appropriate.

Further research should seek to identify whether associative partners (in terms of nearest neighbour) are comparable with relationships identified by the study of interaction partners. Current Secretary of States Standards of Modern Zoo Practice (SSSMZP) elephant management guidelines state that UK and Irish zoos should be providing unique management plans for each elephant and a long-term management plan for the herd including herd compatibility [27]. Current BIAZA elephant management guidelines recommend that UK and Irish zoos should be facilitating the development of positive cohesive relationships between zoos and facilitating fission-fusion dynamics through assessing and responding to individual compatibilities and relationships [30]. The development of reliable metrics to document association patterns and to ascertain whether associate partners are the same as interaction partners in zoo-housed elephants is recommended in order for this data to be incorporated into management. Understanding dyadic relationships has ramifications for individual welfare; in unbalanced social relationships, there is the potential for at least one individual to be experiencing reduced welfare. Regular monitoring of social networks will help to achieve this goal. The British and Irish Association of Zoos and Aquariums (BIAZA) Elephant Welfare Group developed the Elephant Behavioural Welfare Assessment Tool (EBWAT) [56] and health pack that keepers complete on a quarterly basis [27]. The EBWAT includes identification of how frequently (measured on the Likert scale) the elephant has engaged in positive and negative social interactions with other elephants. Keepers could add to this with details on observed social partners, which would enable the incorporation of rudimentary social network analysis into the welfare assessment tool. The temporal dynamics of social relationships would be taken into consideration with data gathered in the EBWAT due to the quarterly nature of observations. Identification of minimum sampling efforts for accurately defining social networks in elephants would also enable the opportunity for keepers to conduct specific social monitoring to incorporate into the herd welfare assessment plan.

Recognition of the fact that animals play different roles in social networks is also extremely important for pro-active management which optimises individual welfare. Monitoring subtle behavioural change has important ramifications for welfare; it provides an opportunity to identify problems and implement mitigation strategies to prevent escalation into more serious long-term issues. Provision of the opportunity for choice of social partners under management regimes that allow social species to show natural behavioural flexibility has been proven to be positive for welfare in zoo chimpanzees [40] and so this should also be considered further as a management tool for other social species, including elephants. Social management decisions must be both practical, in terms of how facilities can manage animals and adherence to breeding programme recommendations and driven by the welfare requirements of individual animals.

## 5. Conclusions

Using elephants as a case study, the aim of this research was to investigate fluidity in social relationships within a zoo environment. Positive networks were more interlinked than negative networks. Temporal dynamics were observed in social networks, but they did not follow a seasonal pattern. Positive networks tended to include the entire social group whereas negative interactions were restricted to specific individuals. There were many unbalanced ties within dyads, suggesting potential inequalities in relationships. This could impact on individual experiences and welfare within the social group. Subtle species-level differences were observed, which leads to a necessity to further understand zoo elephant social behaviour to facilitate evidence-based social management.

This research highlights changing dynamics in animal social networks and provides evidence for the importance of understanding social networks and social behaviour over longer periods of time. Understanding animal social networks is extremely important for evidence-based management, which ensures positive welfare for individuals. Being able to monitor relationships and identify problems before they escalate is necessary within zoos, when animals may have reduced opportunity to escape conflict or a lack of opportunity for choice of social partners. Instability in social networks can have negative effects on welfare experiences in social groups. Understanding social networks provides opportunities for zoos to make the most appropriate decisions when animals are being moved for breeding programmes, to ensure minimal effects on group stability. Information on social networks should be incorporated into animal management guidelines in order to maximise animal welfare. The findings from this research can be applied to inform social management of other zoo-housed species. Further research should seek to identify minimum sampling efforts for social networks in a range of species, to enable execution of regular monitoring of social networks and thus improve the welfare of social species in zoos.

## Figures and Tables

**Table 1:** Elephant and herd demographics for the study elephants at the onset of the study period (October 2015).

Zoo	Elephant	Species	Sex	Age	No. Relatives in Herd	Wild or Captive Born	If Zoo Born, at Natal Zoo?	Observation Period (mins)	Proportion Observations in Sight
A	E1	African	F	45	0	Wild	N/A	5817	0.66
	E2	African	F	47	0	Wild	N/A	5817	0.98
B	E3	Asian	F	54	0	Wild	N/A	5842	0.89
	E4	Asian	F	44	0	Wild	N/A	5842	0.89
	E5	Asian	F	40	0	Wild	N/A	5842	0.85
C	E6	Asian	F	49	0	Captive	N	5838	0.75
	E7	Asian	M	15	1	Captive	N	5838	0.16
	E8	Asian	F	1	4	Captive	Y	5838	0.90
	E9	Asian	F	36	3	Wild	N/A	5838	0.78
	E10	Asian	F	19	3	Captive	Y	5838	0.87
	E11	Asian	F	13	3	Captive	Y	5838	0.87
D	E12	African	M	34	0	Wild	N/A	7666	0.20
	E13	African	F	35	0	Wild	N/A	7666	0.27
	E14	African	F	35	0	Wild	N/A	7666	0.67
	E15	African	F	31	0	Wild	N/A	7666	0.69
E	E16	Asian	F	32	8	Captive	N	3267	0.65
	E17	Asian	F	26	8	Captive	N	3267	0.66
	E18	Asian	F	13	8	Captive	N	3267	0.71
	E19	Asian	F	10	8	Captive	Y	3267	0.75
	E20	Asian	M	2	9	Captive	Y	3267	0.61
	E21	Asian	F	2	9	Captive	Y	3267	0.65
	E22	Asian	M	2	9	Captive	Y	3267	0.60
	E23	Asian	F	<1	9	Captive	Y	1569	0.51
	-	Asian	M	22	9	Captive	N		
F	E24	African	F	14	1	Captive	Y	5031	0.79
	E25	African	F	30	0	Wild	N/A	5031	0.76
	E26	African	F	14	2	Captive	Y	5031	0.81
	E27	African	F	30	1	Wild	N/A	5031	0.80
G	E28	Asian	F	33	0	Wild	N/A	5016	0.69
	E29	Asian	F	22	1	Captive	N	5016	0.70
	E30	Asian	F	3	1	Captive	Y	5016	0.63
	E31	Asian	F	19	1	Captive	Y	5016	0.68
	E32	Asian	F	34	1	Wild	N/A	5016	0.67

No social behaviour data was available for the bull elephant at Zoo E due to video camera quality from outside enclosures. He was therefore removed from the study.

**Table 2:** Data collection periods for each study zoo.

Zoo	Data Collection Period (Study Months, Days)			
	1	2	3	4
A	January and February 2016 (10 days)	April and May 2016 (10 days)	July and August 2016 (10 days)	October and November 2016 (10 days)
B	May 2016 (5 days)	August 2016 (5 days)	December 2016 (5 days)	February 2017(5 days)
C	January and February 2016 (10 days)	April and May 2016 (10 days)	July and August 2016 (10 days)	October and November 2016 (10 days)
D	January and February 2016 (10 days)	April and May 2016 (10 days)	July and August 2016 (10 days)	October and November 2016 (10 days)
E	February 2016	April and May 2016	September 2016	October and November 2016
F	January and February 2016 (10 days)	April and May 2016 (10 days)	July and August 2016 (10 days)	October and November 2016 (10 days)
G	January and February 2016 (10 days)	April and May 2016 (10 days)	July and August 2016 (10 days)	October and November 2016 (10 days)

**Table 3:** Elephant behaviour ethogram [32].

Behaviour			Description
Positive	Positive physical	Conspecific play	Engaging in active play with another elephant, including head-to-head sparring, trunk wrestling, mounting, chasing and rolling on one another. Does not include behaviours observed following an agonistic encounter or courtship.
		Touching (trunk to)	Touching another elephant with the trunk in a non-aggressive manner.
		Touching (body to)	Touching/rubbing another elephant with the body.
	Positive non-physical	Protecting	Standing over another elephant.
		Huddling	Formation of a tight circle with calves at the nucleus. Calves hidden in the middle, adults surrounding them.
		Approach	Walking towards another elephant in a non-threatening manner. Recipient stays in position during and after the approach.
		Approach with trunk	Trunk outstretched towards another elephant. Not close enough to make physical contact.
		Walking with	Walking side by side with another elephant.
		Following	Walking closely behind another elephant (within one elephant body length).
Negative	Negative physical	Pushing	One elephant forces or pushes against the body (usually the rump) of another elephant, resulting in the elephant that is being pushed moving at least two steps.
		Pulling	Using the trunk to pull at another elephant in a non-playful manner. May pull at the trunk or an accessible body part such as tusks/tushes or the tail.
		Sparring	An escalation of a push/pull incident into more physical aggression.
		Hitting/kicking	Aggressive physical contact with the trunk or leg, e.g., trunk strike or kicking out.
	Negative non-physical	Displace	Movement of one elephant results in another elephant leaving its location (within 10 s)—usually occurs when a more dominant elephant approaches a more subordinate individual.
		Approach	Walking towards another elephant in an aggressive or hostile manner (head held high, ears wide or flapping). Receiving elephant may either respond to this by standing as tall as possible, head raised, ears flapping or turning away from/walking away from the approaching elephant.
		Walking/turning away from	Avoiding or shying away from elephants or people; the individual either walks forwards away from or backwards away from a particular elephant or person.
		Frozen	Standing still and alert as another elephant approaches.
		Charge/mock charge	Move towards another elephant with the head held high, pace usually quickens as the individual gets closer to the target elephant. In the case of a mock charge, the individual charging stops further away from the target elephant.
		Blocking	Blocking from food source or other resource (e.g., door)

**Table 4:** Median recorded frequencies of social interactions given by each study elephant.

Interaction Type	Time Period	Median	IQR	Range (%)
Positive physical	1	0.96	0.09–4.28	0–8.55
	2	0.19	0.19–5.3	0–18.27
	3	0.86	0.86–4.77	0–14.03
	4	1.16	1.16–6.56	0–13.73
Negative physical	1	0	0–0.05	0–0.3
	2	0.02	0–0.07	0–0.19
	3	0.02	0–0.04	0–0.16
	4	0	0–0.06	0–0.48
Positive non-physical	1 *^, 2,3,4^	3.35	3.35–8.19	0.13–50.65
	2 *^, 1,3,4^	1.57	1.57–6	0.03–16.84
	3 *^, 1,2^	1.04	1.04–1.96	0–11.34
	4 *^, 1,2^	1.29	1.29–2.24	0.15–6.52
Negative non-physical	1	0.19	0.06–0.34	0–1.1
	2	0.09	0.04–0.23	0–0.85
	3	0.09	0.03–0.24	0–3.28
	4	0.06	0.03–0.18	0–0.52

* Indicates a significant difference. The number in superscript ^1, 2, 3, 4^ indicates in which time period the significant differences occurred. IQR: interquartile range.

**Table 5:** Median recorded frequencies of social interactions given within elephant dyads.

Interaction Type	Time Period	Median	IQR	Range (%)
Positive physical	P1 *^,4^	0	0–0.15	0–7.52
	P2	0	0–0.07	0–12.01
	P3	0	0–0.16	0–8.89
	P4 *^,1^	0.03	0–0.71	0–11.65
Negative physical	P1	0	0–0	0–0.23
	P2	0	0–0	0–0.15
	P3	0	0–0	0–0.09
	P4	0	0–0	0–0.48
Positive non-physical	P1 *^,2,3,4^	0.27	0–0.97	0–17.16
	P2 *^,1,3^	0.13	0–0.53	0–10.81
	P3 *^,1,2,4^	0.07	0–0.27	0–10.81
	P4 *^,1,3^	0.13	0.12–0.31	0–6.14
Negative non-physical	P1 *^, 2,3,4^	0	0–0.06	0–0.76
	P2 *^,1^	0	0–0.04	0–0.85
	P3 *^,1^	0	0–0.03	0–3.28
	P4 *^,1^	0	0–0.03	0–0.44

* Indicates a significant difference. The number in superscript ^1, 2, 3, 4^ indicates in which time period the significant differences occurred. IQR: interquartile range.

**Table 6:** Mantel test correlation scores showing dyadic reciprocity in the study herds.

Zoo	Physical		Non-Physical	
	Positive	Negative	Positive	Negative
A	N/A	N/A	N/A	N/A
B	NS	NS	NS	NS
C	0.8455 *	NS	0.8965 **	0.8551 *
D	NS	NS	NS	NS
E	0.5341 **	NS	NS	0.6821 **
F	0.9761 *	NS	NS	NS
G	0.9348 *	NS	NS	NS

N/A: No physical interactions were observed at Zoo A. Mantel test statistics could not be performed on the data entered for non-physical interactions. Significance values are indicated by * *p* < 0.05, ** *p* < 0.01.

**Table 7:** Mantel test correlation scores showing stability over time for social interactions in the study herds.

Interaction Type	Comparison Points	Zoo						
		A	B	C	D	E	F	G
Positive physical	1	N/A	NS	0.9834 **	NS	NS	NS	0.9204 ***
	2	N/A	NS	NS	NS	NS	0.8279 *	NS
	3	N/A	NS	NS	NS	NS	NS	NS
	4	N/A	NS	1 **	NS	NS	NS	NS
	5	N/A	NS	NS	NS	0.7266 **	NS	NS
	6	N/A	NS	0.9897 *	NS	NS	NS	NS
Positive non-physical	1	N/A	NS	NS	NS	NS	NS	0.9206 *
	2	N/A	NS	NS	NS	NS	NS	0.8353 *
	3	N/A	NS	0.7289 *	NS	NS	0.9113 *	0.9444 *
	4	N/A	NS	0.8054 **	NS	0.713 **	NS	0.8622 **
	5	N/A	NS	NS	NS	0.6924 *	NS	0.8118 **
	6	N/A	NS	0.9704 **	NS	NS	0.9113 *	0.7876 *
Negative physical	1	N/A	N/A	0.6784 *	N/A	NS	N/A	NS
	2	N/A	NS	0.8688 **	N/A	N/A	N/A	NS
	3	N/A	NS	0.93 **	N/A	N/A	NS	NS
	4	N/A	N/A	0.7917 **	N/A	N/A	NS	NS
	5	N/A	NS	NS	N/A	NS	NS	NS
	6	N/A	NS	0.9376 **	N/A	NS	NS	NS
Negative non-physical	1	N/A	NS	0.6346 *	NS	NS	NS	NS
	2	N/A	NS	0.6478 *	NS	NS	NS	NS
	3	N/A	NS	0.5476 *	NS	NS	NS	NS
	4	N/A	NS	NS	NS	0.591 *	NS	NS
	5	N/A	NS	NS	NS	NS	NS	NS
	6	N/A	NS	0.6828 *	NS	NS	NS	NS

N/A: Physical negative interactions could not be analysed due to no occurrence of these interactions in one of the matrices. Mantel tests were not calculated for Zoo A due to the death of E2 following the first month of data collection. Significance values are indicated by * *p* < 0.05, ** *p* < 0.01 and *** *p* < 0.001.

**Table 8:** Betweenness centrality scores for study elephants.

Zoo	Elephant	Species	Sex	Age	Related to Others in Herd	Betweenness Score			
						Positive Physical	Positive Non-Physical	Negative Physical	Negative Non-Physical
A	E1 ^M^	African	F	45	N	N/A	0	N/A	0
	E2	African	F	47	N	N/A	0	N/A	0
B	E3 ^M^	Asian	F	54	N	1	0	0	0
	E4	Asian	F	44	N	0	0	0	0
	E5	Asian	F	40	N	0	0	0	0
C	E6	Asian	F	49	N	0	0	0	0.25
	E7	Asian	M	15	Y	0	0	0	0
	E8 ^M^	Asian	F	36	Y	0	0	0	0.25
	E9	Asian	F	1	Y	0	0	0	0
	E10	Asian	F	19	Y	0	0	0	0.25
	E11	Asian	F	13	Y	0	0	0	0.25
D	E12 ^M^	African	M	34	N	0	0	0.5	0
	E13	African	F	35	N	0	0	0	0
	E14 ^M^	African	F	35	N	0	0	0.5	0
	E15	African	F	31	N	0	0	0	0
E	E16 ^M^	Asian	F	32	Y	0	0	6	0.25
	E17	Asian	F	26	Y	0.4	0	0	0.67
	E18	Asian	F	13	Y	0.4	0	6.33	0.92
	E19	Asian	F	10	Y	0	0	2	0.25
	E20	Asian	M	2	Y	0.4	0	0.33	0.67
	E21	Asian	F	2	Y	0.4	0	7	0.25
	E22	Asian	M	2	Y	0.4	0	3.33	0
	E23	Asian	F	<1	Y	0	0	0	0
F	E24	African	F	14	Y	0	0	0	0
	E25 ^M^	African	F	30	N	0	0	0	0
	E26	African	F	14	Y	0	0	0	0
	E27	African	F	30	Y	0	0	0	0
G	E28 ^M^	Asian	F	33	N	0	0	0	0
	E29	Asian	F	22	Y	0	0	0	0
	E30	Asian	F	3	Y	0	0	0	0
	E31	Asian	F	19	Y	0	0	0	0
	E32	Asian	F	34	Y	0	0	0	0

^M^ denotes the matriarch or elephant considered to be the most dominant herd member.

**Table 9:** Dyadic relationships considered to be balanced (assessed using simple ratios) in the study herds.

Zoo	Physical Positive		Physical Negative		Non-Physical Positive		Non-Physical Negative	
**A**								
**B**								
C	E6–E10 E7–E8 E8–E9	Unrelated Unrelated Related	E6–E10 E6–E11 E7–E8 E7–E9	Unrelated Unrelated Unrelated Related	E6–E8 E6–E9 E6–E10 E6–E11 E7–E8 E7–E10 E9–E10 E10–E11	Unrelated Unrelated Unrelated Unrelated Related Unrelated Related Related	E6–E9 E6–E10 E10–E11	Unrelated Unrelated Related
D					E14–E15	Unrelated	E14–E15	Unrelated
E	E19–E21 E18–E22 E20–E21	Related Related Related	E16–E17	Related	E16–E17 E16–E18 E17–E19 E18–E19 E21–E23 E22–E23	Related Related Related Related Related Related	E16–E21 E17–E19 E17–E20	Related Related Related
F	E24–E25 E26–E27	Unrelated Related	E24–E25	Unrelated	E24–E25 E24–E26 E24–E27 E25–E27	Unrelated Related Unrelated Unrelated	E24–E25 E26–E27	Unrelated Related
G	E28–E30 E28–E31 E28–E32 E29–E30 E29–E31 E30–E31	Unrelated Unrelated Unrelated Related Unrelated Unrelated			E28–E29 E28–E31 E29–E31 E30–E32	Unrelated Unrelated Unrelated Unrelated	E29–E31	Unrelated

## Supplementary Materials

The following are available online at https://www.mdpi.com/2076-2615/10/5/882/s1, Figure S1. Sociograms depicting (a) non-physical positive interactions (b) non-physical negative interactions recorded at Zoo A, Figure S2. Sociograms depicting (a) physical positive interactions and (b) non-physical positive interactions at Zoo B, Figure S3. Sociograms depicting (a) physical negative interactions and (b) non-physical negative interactions recorded at Zoo B, Figure S4. Sociograms depicting (a) physical positive interactions and (b) non-physical positive interactions at Zoo C, Figure S5. Sociograms depicting (a) physical negative interactions and (b) non-physical negative interactions at Zoo C, Figure S6. Sociograms depicting (a) physical positive interactions and (b) non-physical positive interactions at Zoo D, Figure S7. Sociograms depicting (a) physical negative interactions and (b) non-physical negative interactions at Zoo D, Figure S8. Sociograms depicting (a) physical positive interactions and (b) non-physical positive interactions at Zoo E, Figure S9. Sociograms depicting (a) physical negative interactions and (b) non-physical negative interactions at Zoo E, Figure S10. Sociograms depicting (a) physical positive interactions and (b) non-physical positive interactions at Zoo F, Figure S11. Sociograms depicting (a) physical negative interactions and (b) non-physical negative interactions at Zoo F, Figure S12. Sociograms depicting (a) physical positive interactions and (b) non-physical positive interactions at Zoo G, Figure S13. Sociograms depicting (a) physical negative interactions and (b) non-physical negative interactions at Zoo G.
