# Supplementary material for: Understanding Temporal Social Dynamics in Zoo Animal Management: An Elephant Case Study
Source: Animals (Basel). 2020 May 19;10(5):882. doi: 10.3390/ani10050882 (PMC7278397; doi:10.3390/ani10050882)
Supplement: Supplementary file 1 [file animals-10-00882-s001.pdf]

animals

MDPI

Supplementary material

Understanding Temporal Social Dynamics in Zoo Animal Management: An Elephant Case Study

Sociograms for each of the study zoos are provided in Figures S1 to S14. In all instances interaction frequency is depicted by line thickness. Numbers next to nodes are indicative of the mean percentage of interactions received from the corresponding elephant. The centrality of individuals within networks was used to quantify the importance of individuals within the social groups [1]. This study dealt with directed networks using interaction data in order to measure how important an individual was in terms of cohesion of overall herd structure. Betweenness centrality was considered to provide the most useful and relevant information. Betweenness centrality scores for each individual elephant are presented beneath the sociograms. A higher value indicates a greater influence within the social group.

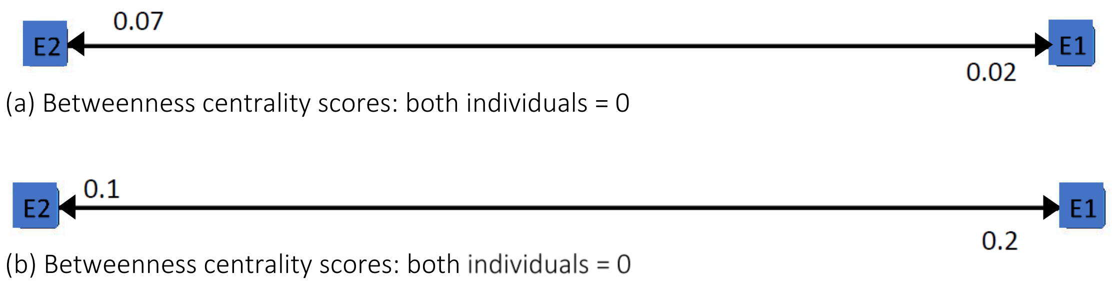

Figure S1. Sociograms depicting (a) non-physical positive interactions (b) non-physical negative interactions recorded at Zoo A. A higher number of non-physical positive interactions were given by the matriarch. Non-physical negative interactions were approximately equal.

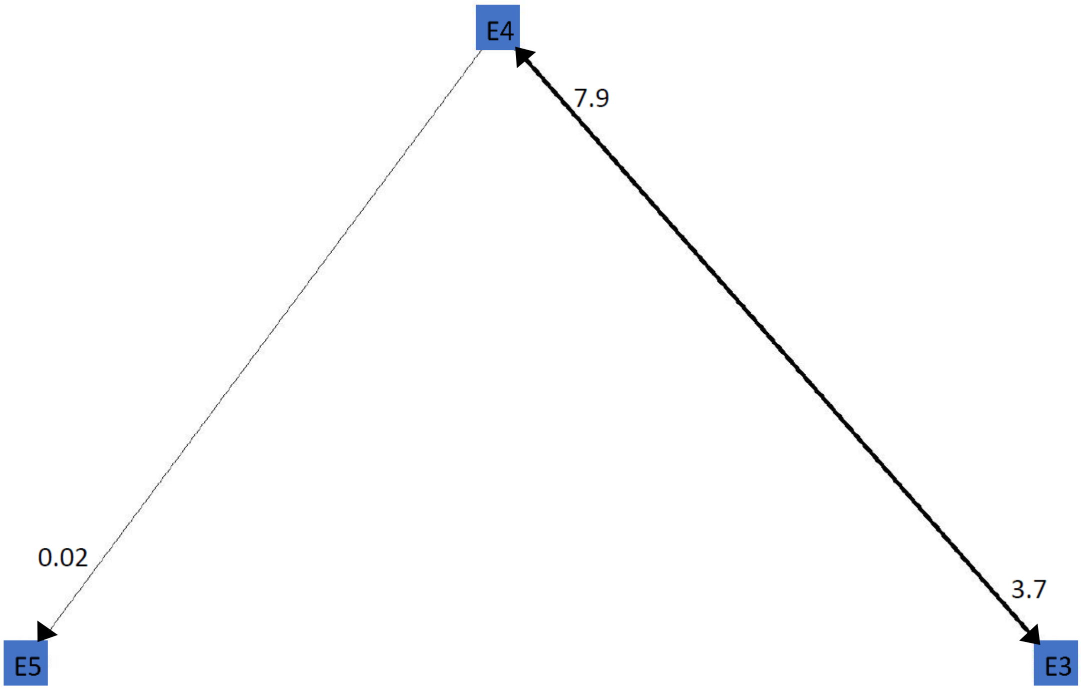

(a) Betweenness centrality scores: E3 = 1, E4 = 0, E5 = 0

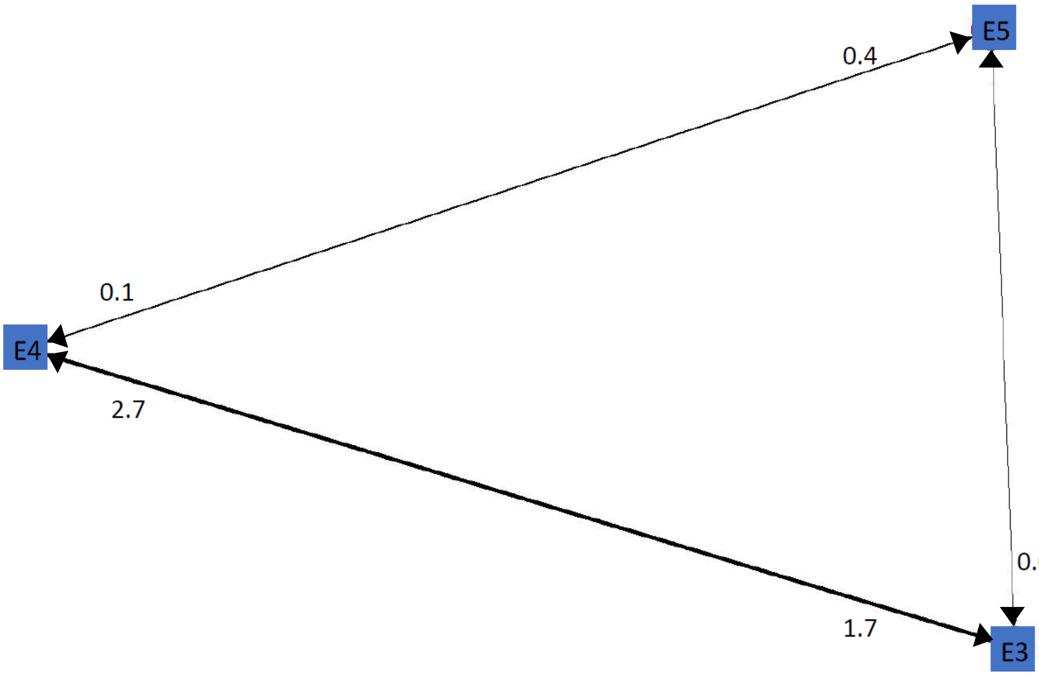

(b) Betweenness centrality scores: all individuals = 0

Figure S2. Sociograms depicting (a) physical positive interactions and (b) non-physical positive interactions at Zoo B. E4, considered by keepers to be matriarch, was most central to the positive physical network (identified via highest betweenness score). Betweenness scores were equal across the group for the positive non-physical network. E5 gave no physical interactions during the study.

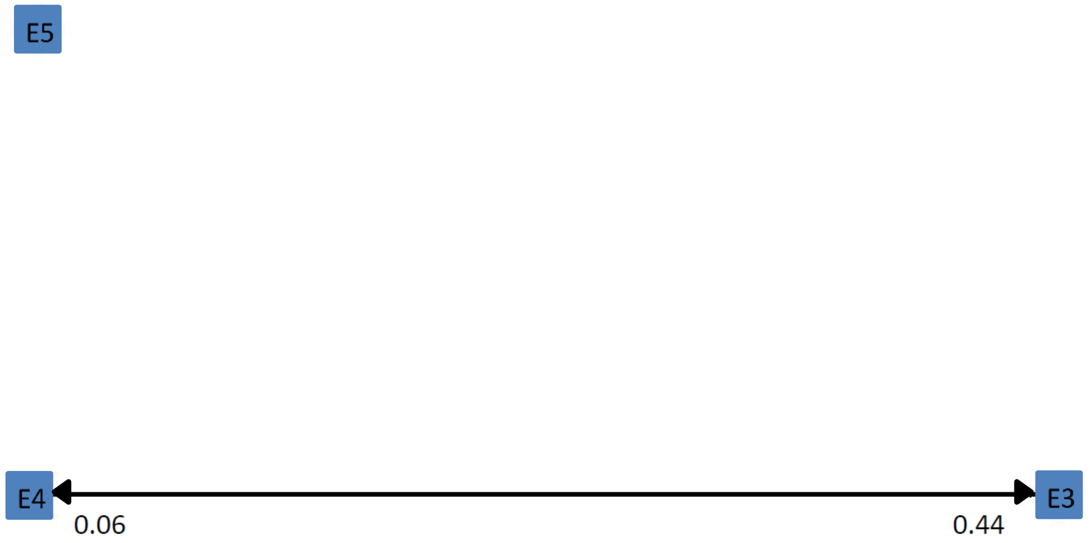

(a) Betweenness centrality scores: all individuals = 0

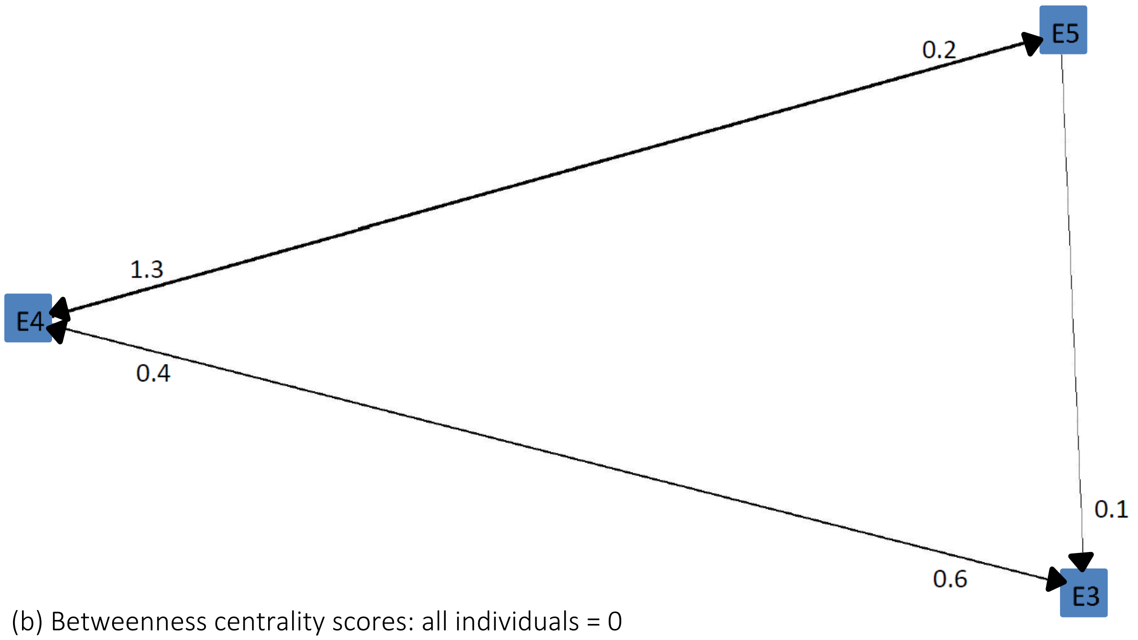

(b) Betweenness centrality scores: all individuals = 0

Figure S3. Sociograms depicting (a) physical negative interactions and (b) non-physical negative interactions recorded at Zoo B. Betweenness scores were equal across the group for both networks. E5 gave no physical negative interactions during the study.

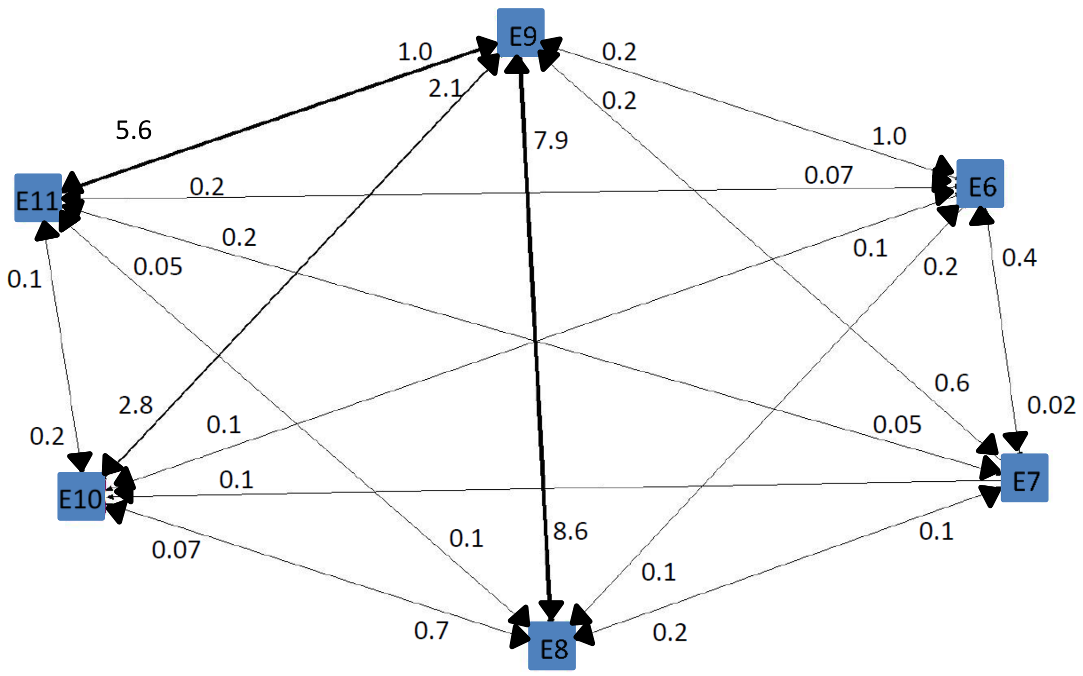

(a) Betweenness centrality scores: all individuals = 0

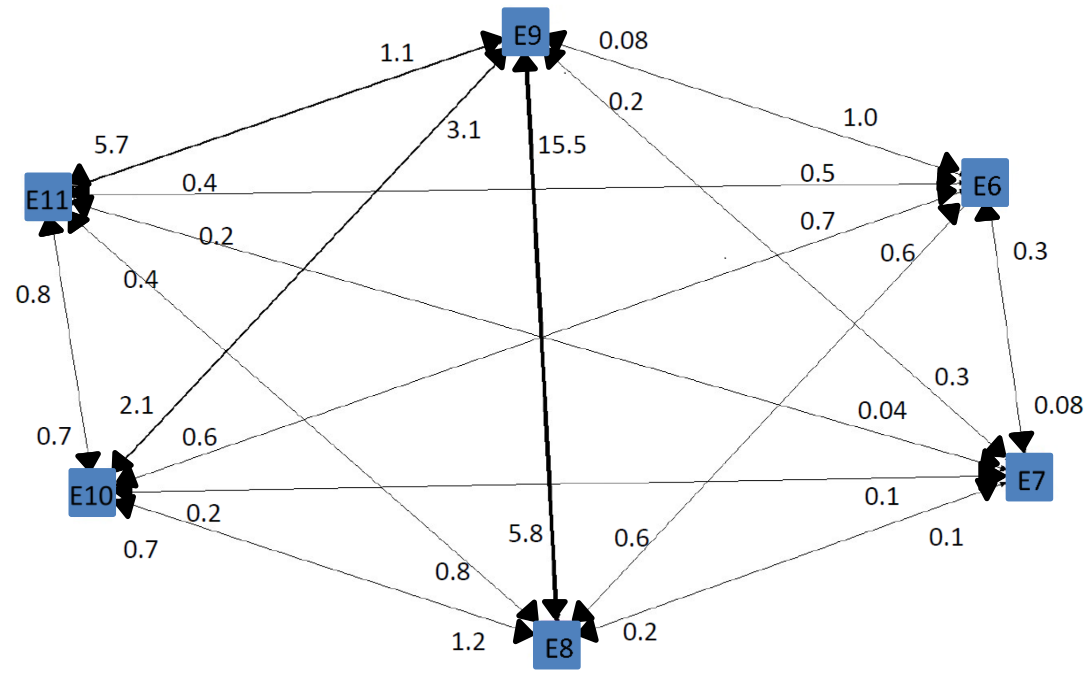

(b) Betweenness centrality scores: all individuals = 0

Figure S4. Sociograms depicting (a) physical positive interactions and (b) non-physical positive interactions at Zoo C. The greatest number of physical and non-physical interactions were between the matriarch and her calf (E8 & E9) although betweenness scores were equal across the group.

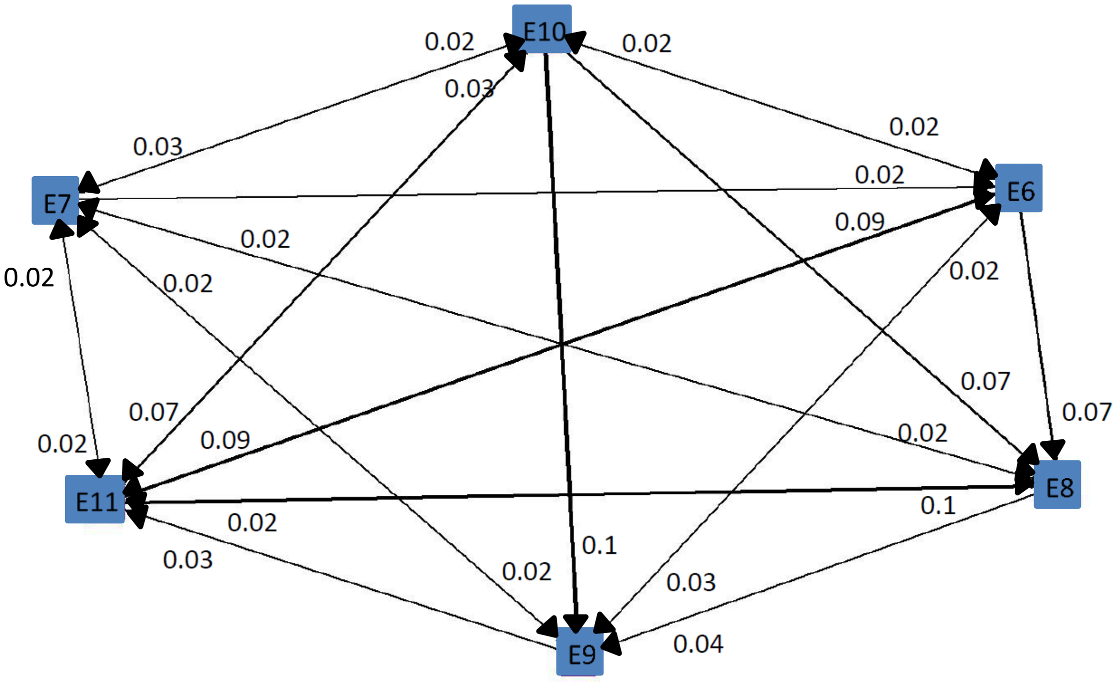

(a) Betweenness centrality scores: all individuals = 0

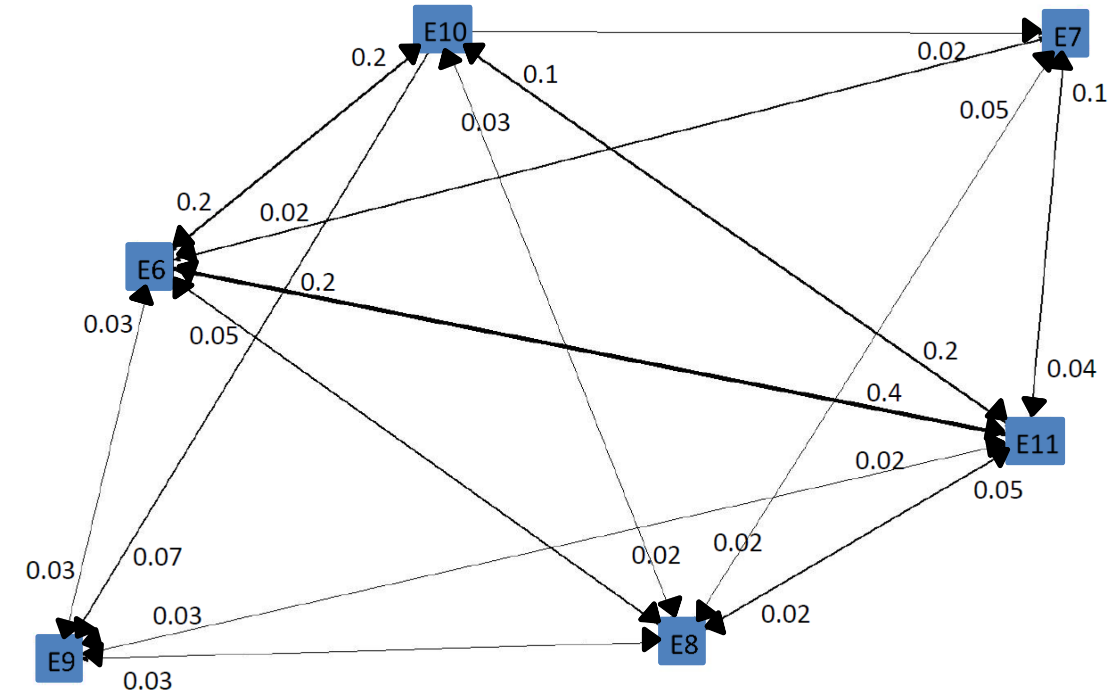

(b) Betweenness centrality scores: E6 = 0.25, E7 = 0, E8 = 0.25, E9 = 0, E10 = 0.25, E11 = 0.25

Figure S5. Sociograms depicting (a) physical negative interactions and (b) non-physical negative interactions at Zoo C. Negative physical interactions were fairly low and evenly spread throughout the group (equal betweenness scores across all individuals). Negative non-physical interactions were greatest between E6 and E11, unrelated adult females. E6, E8, E10 and E11 were considered most central to the network (indicated via highest betweenness scores).

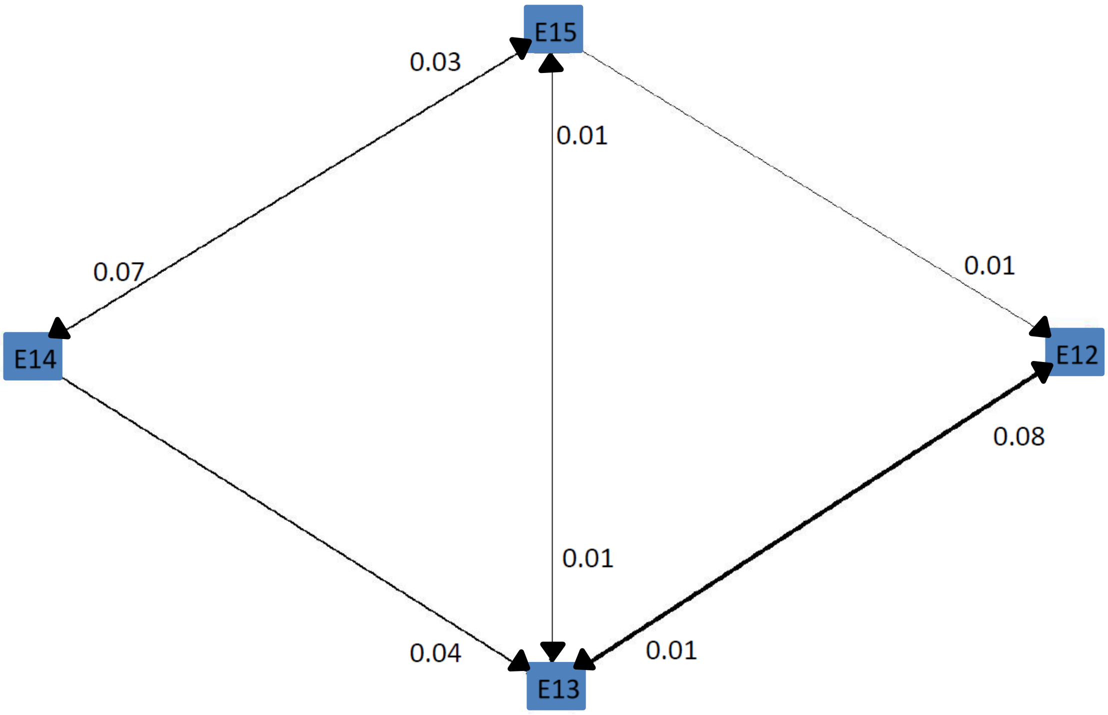

(a) Betweenness centrality scores: all individuals = 0

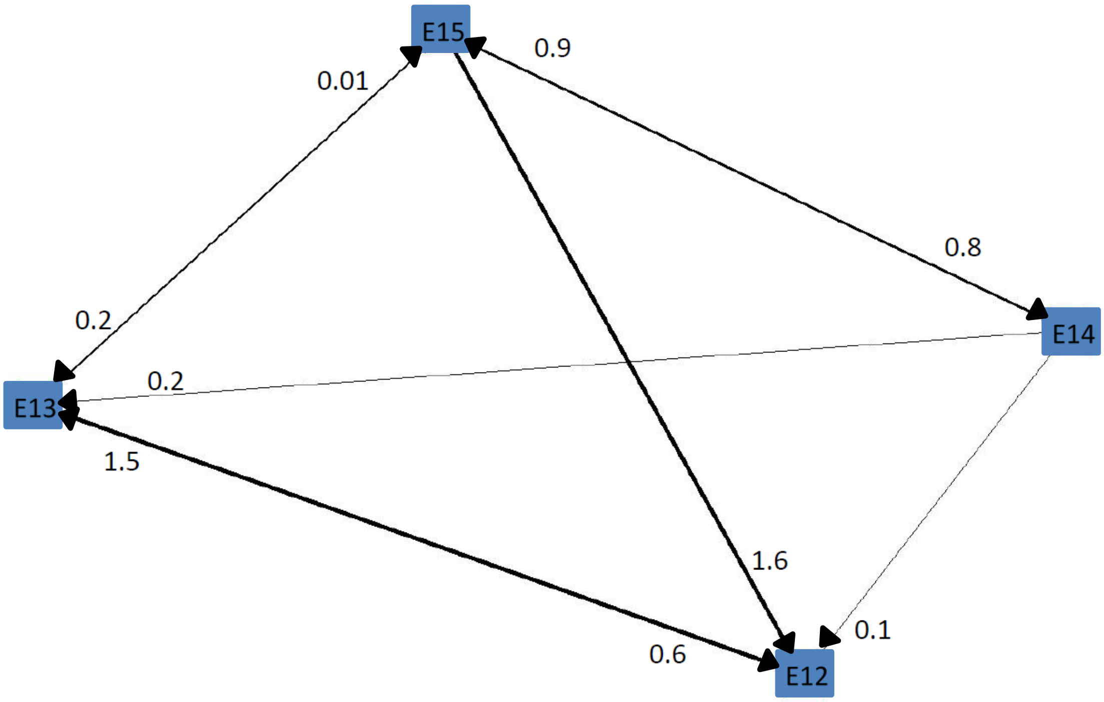

(b) Betweenness centrality scores: all individuals = 0

Figure S6. Sociograms depicting (a) physical positive interactions and (b) non-physical positive interactions at Zoo D. E12 and E13 were housed together during the day and separated overnight – tactile contact was possible through enclosure bars. E14 and E15 were housed together 24/7. E14 and E15 had the opportunity for tactile contact with E12 and E13 through enclosure bars overnight. E12, the adult bull, gave the most non-physical interactions to the female with which he was housed and received most non-physical interactions from another female. E14, the matriarch only received

non-physical positive interactions from the female with which she was housed. Betweenness scores were equal in both networks. .

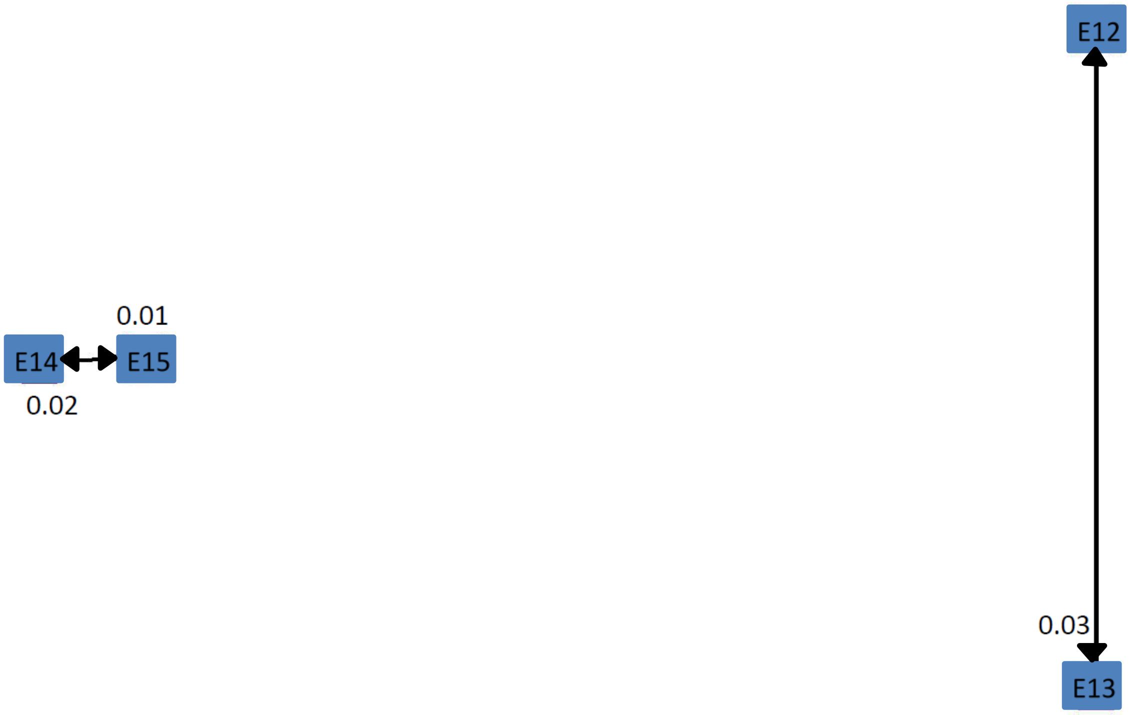

(a) Betweenness centrality scores: E12 = 0.5, E13 = 0, E14 = 0.5, E15 = 0

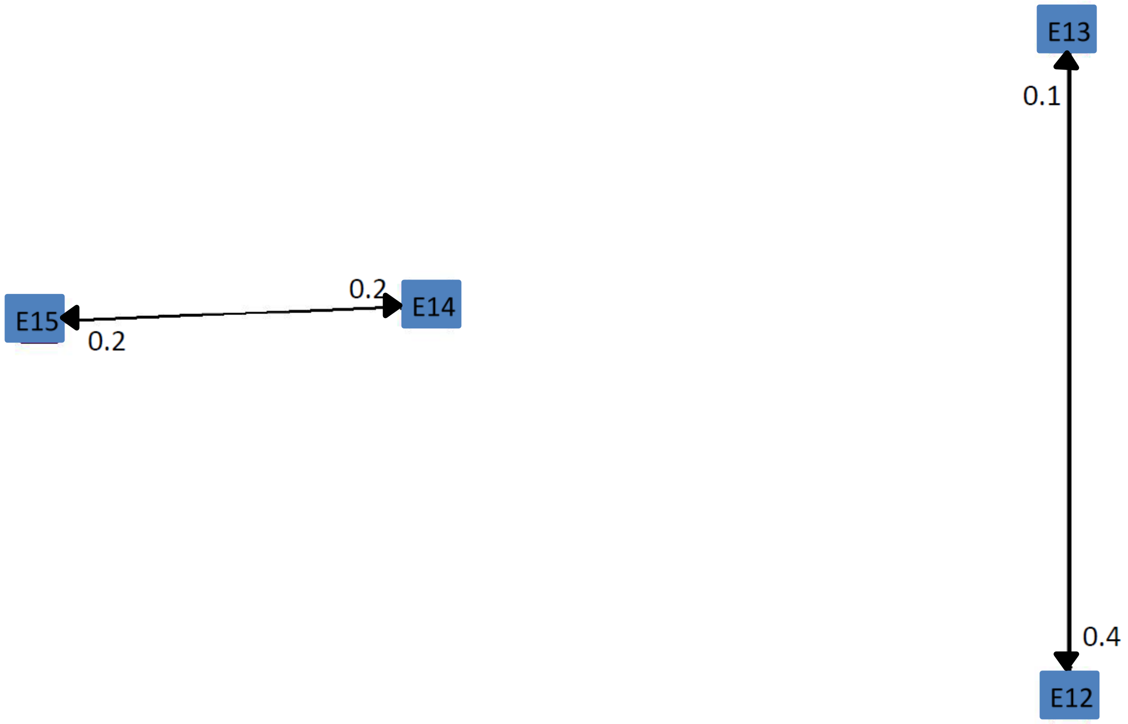

(b) Betweenness centrality scores: all individuals = 0

Figure S7. Sociograms depicting (a) physical negative interactions and (b) non-physical negative interactions at Zoo D. Negative interactions were only observed between elephants housed together. E12 and E14 were considered central to the physical negative network (indicated by highest

betweenness scores). Interactions were equal in the non-physical negative network. Betweenness scores were equal in both networks.

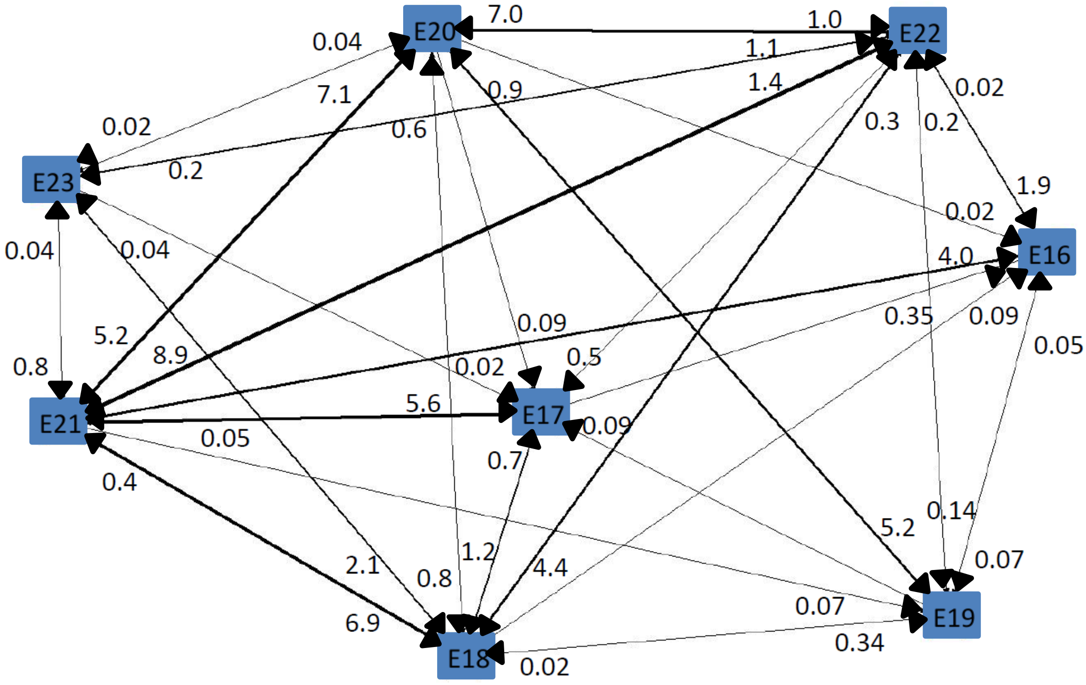

(a) Betweenness centrality scores: E16 = 0, E17 = 0.4, E18 = 0.4, E19 = 0, E20 = 0.4, E21 = 0.4, E22 = 0.4, E23 = 0

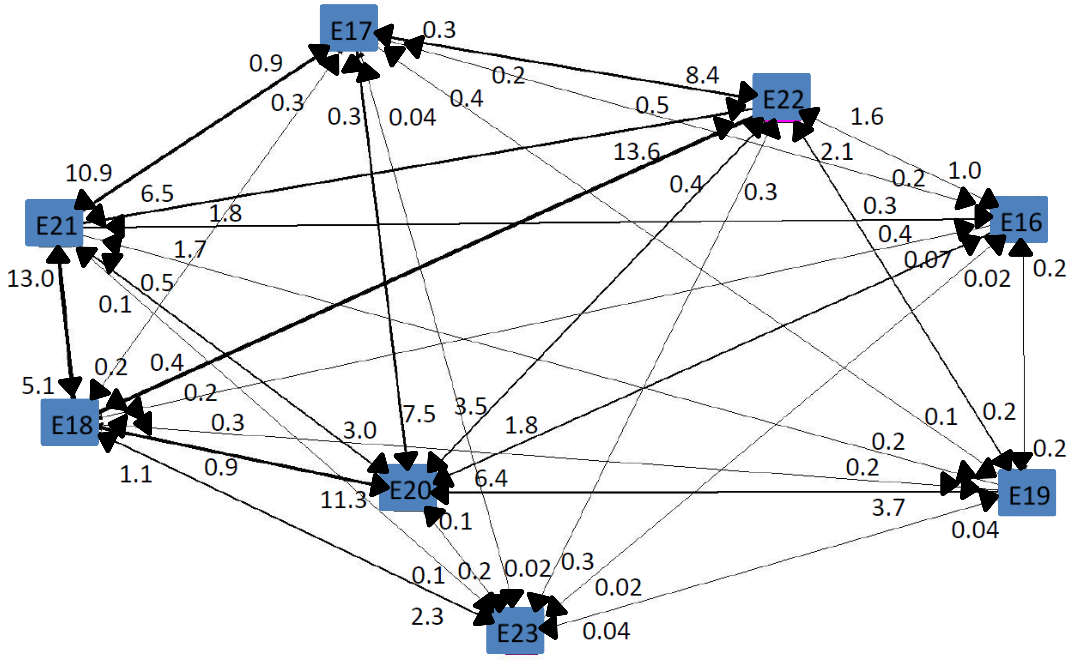

(b) Betweenness centrality scores: all individuals = 0

Figure S8. Sociograms depicting (a) physical positive interactions and (b) non-physical positive interactions at Zoo E. The networks are highly interlinked with calves giving and receiving most interactions. E17, E18, E20, E21 and E22 were central to the positive physical interaction network

(indicated by highest betweenness scores). Non-physical positive interactions were equally distributed.

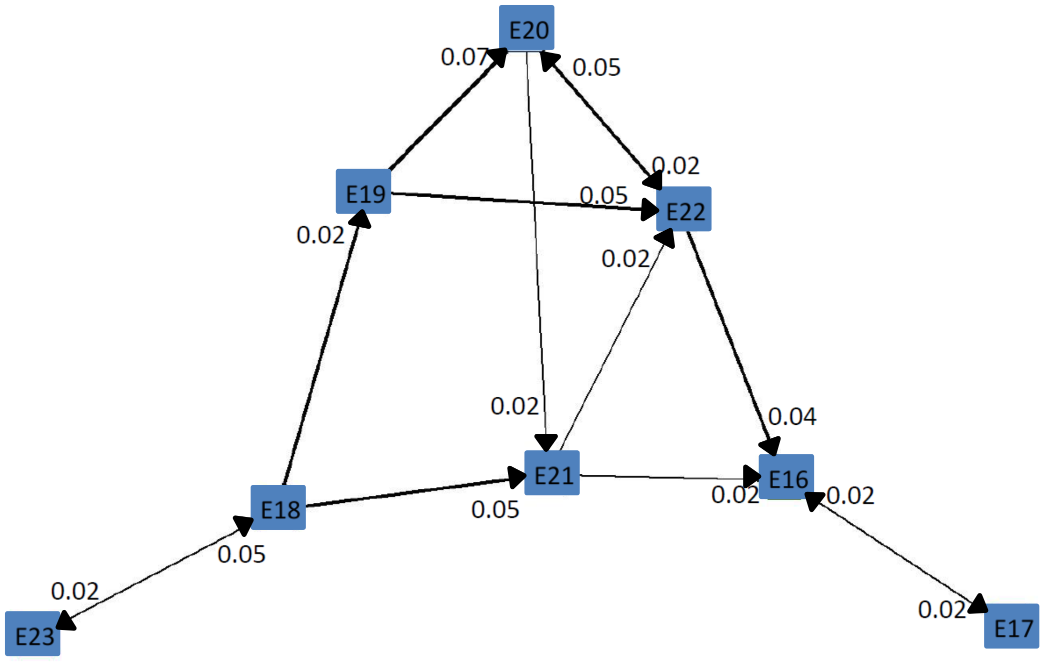

(a) Betweenness centrality scores: E16 = 6, E17 = 0, E18 = 6.33, E19 = 2, E20 = 0.33, E21 = 7, E22 = 3.33, E23 = 0

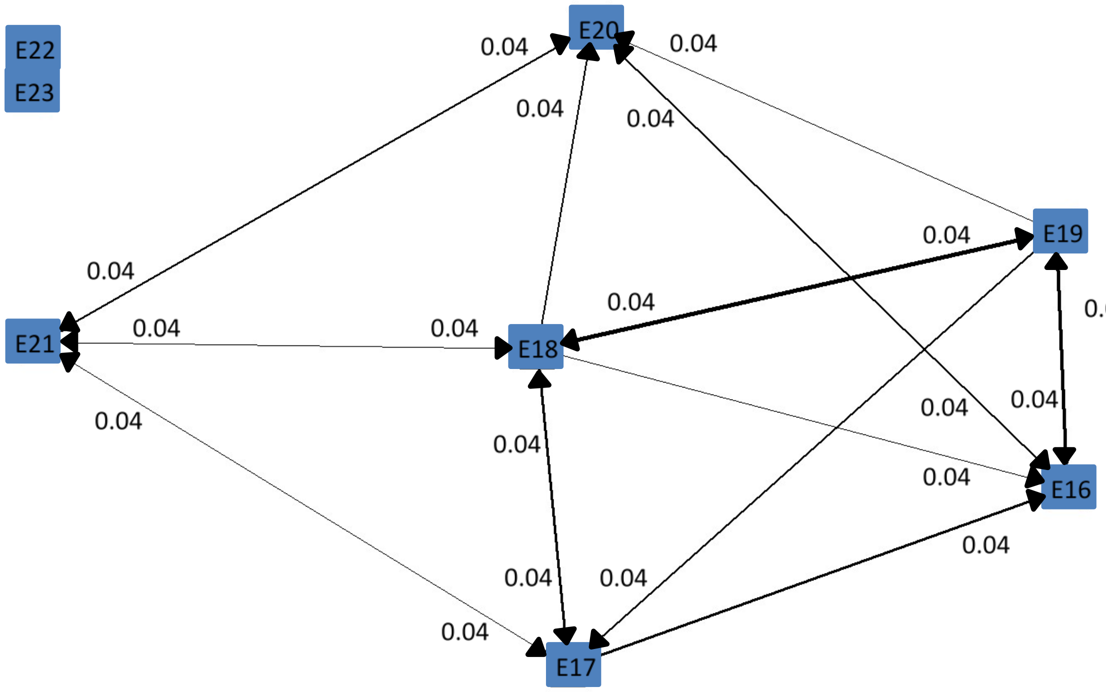

(b) Betweenness centrality scores: E16 = 0.25, E17 = 0.67, E18 = 0.92, E19 = 0.25, E20 = 0.67, E21 = 0.25, E22 = 0, E23 = 0

Figure S9. Sociograms depicting (a) physical negative interactions and (b) non-physical negative interactions at Zoo E. Negative interactions were performed in low frequencies and did not include all members of the group. E21 was most central in the physical negative interaction network and E18 was most central in the non-physical network (indicated by highest betweenness score). E22 and E23 did not give or receive any negative non-physical interactions.

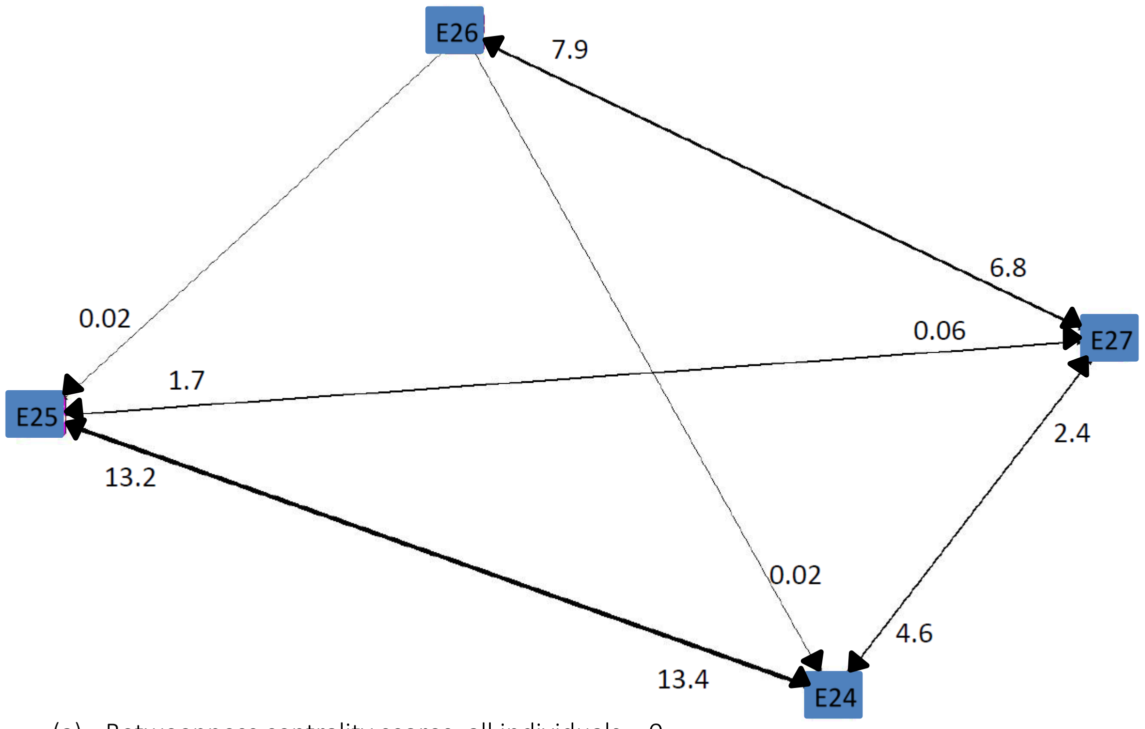

(a) Betweenness centrality scores: all individuals = 0

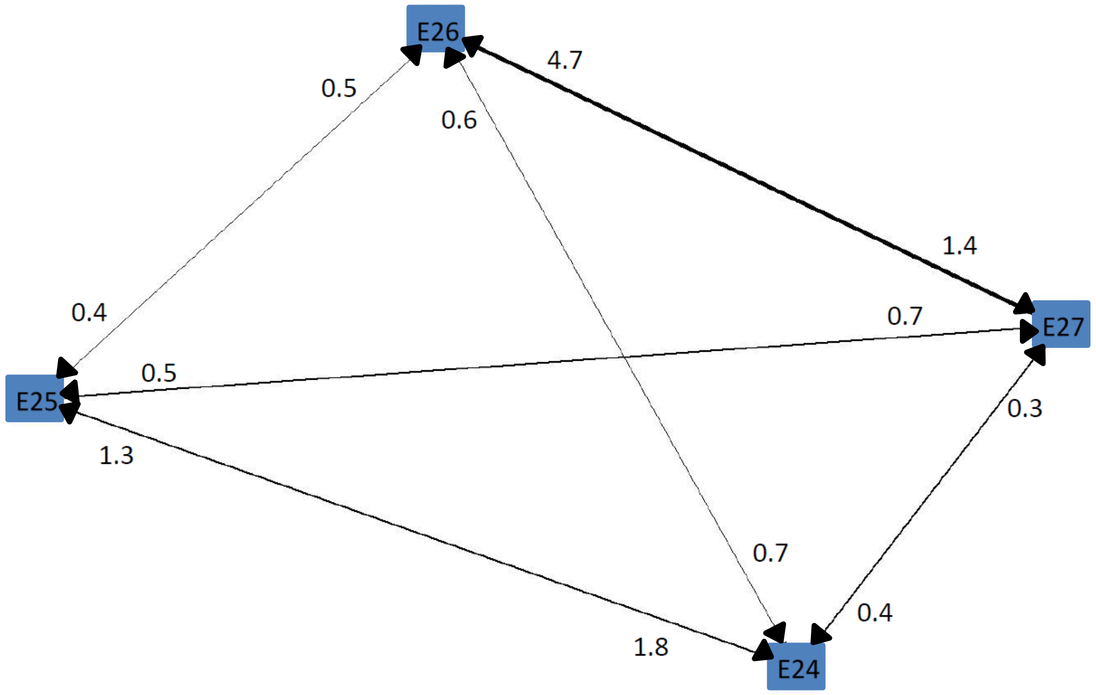

(b) Betweenness centrality scores: all individuals = 0

Figure S10. Sociograms depicting (a) physical positive interactions and (b) non-physical positive interactions at Zoo F. Interactions were greatest between the two dyads (mother/surrogate mother – daughter), interactions were seen across the whole group but they were less frequent. Betweenness scores were equal in both networks.

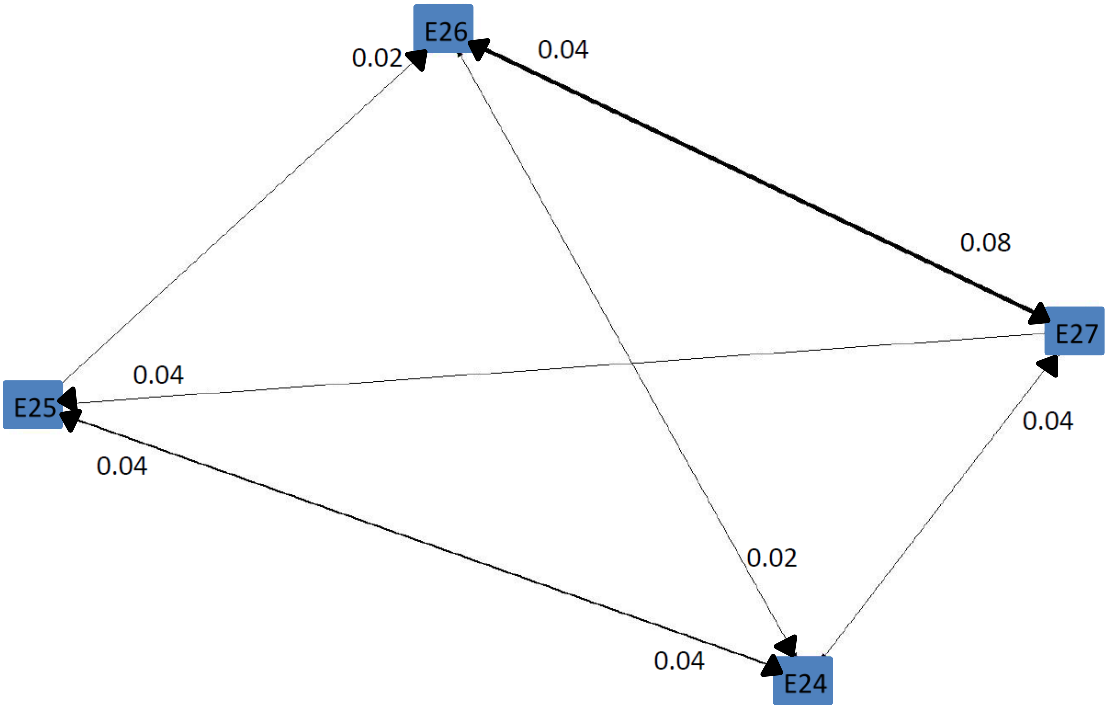

(a) Betweenness centrality scores: all individuals = 0

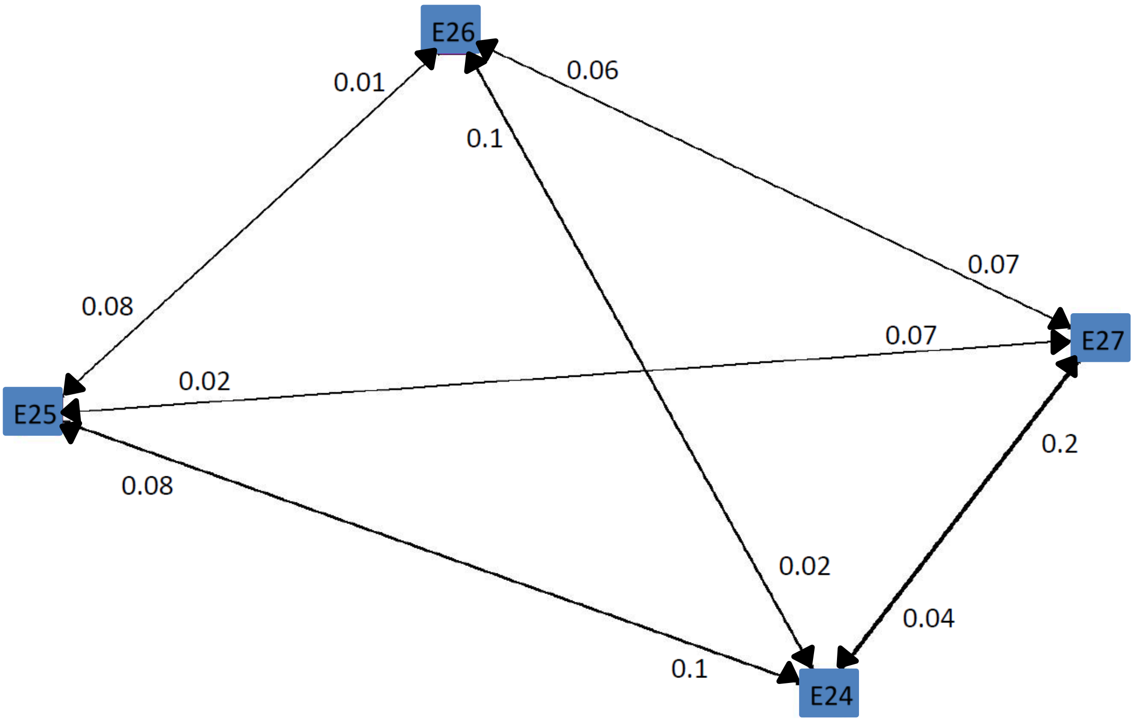

(b) Betweenness centrality scores: all individuals = 0

Figure S11. Sociograms depicting (a) physical negative interactions and (b) non-physical negative interactions at Zoo F. Physical negative interactions were greatest between the two dyads (mother/surrogate mother – daughter). Non-physical negative interactions were more evenly distributed across the group. Betweenness scores were equal in both networks.

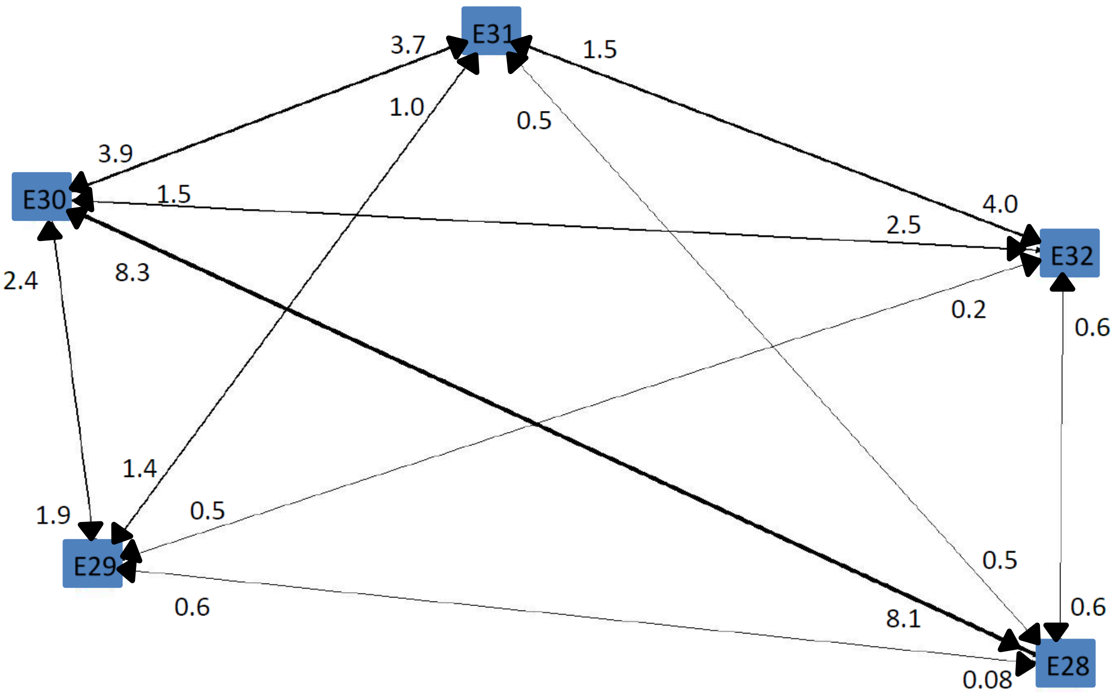

(a) Betweenness centrality scores: all individuals = 0

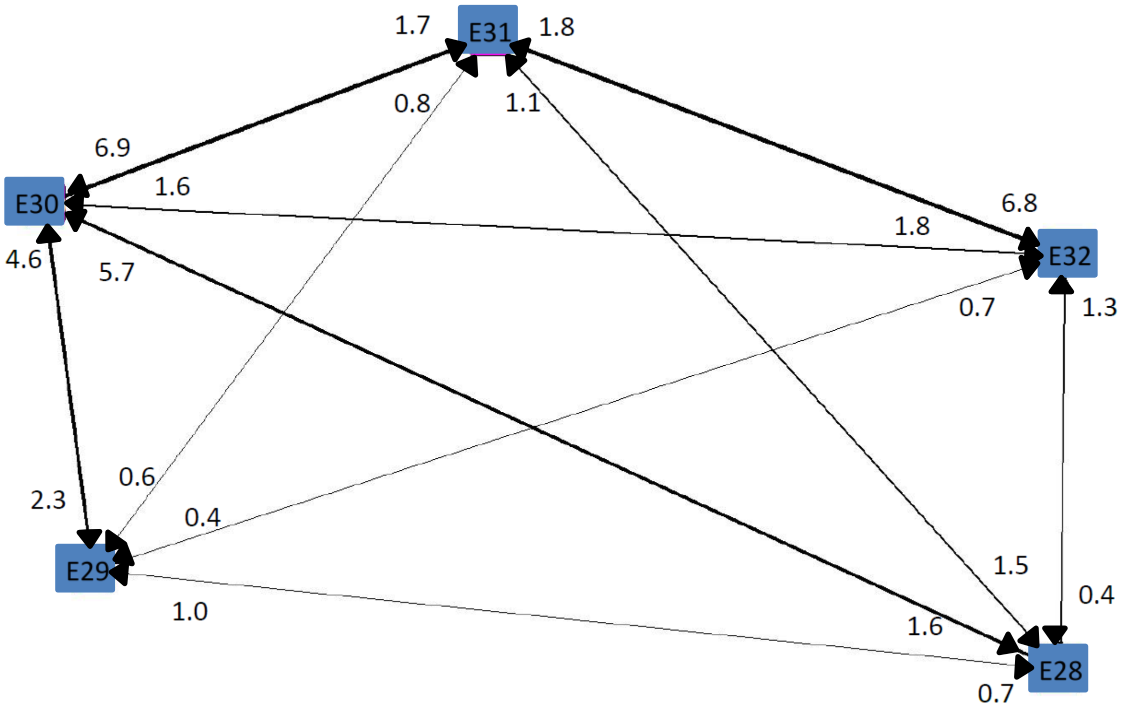

(b) Betweenness centrality scores: all individuals = 0

Figure S12. Sociograms depicting (a) physical positive interactions and (b) non-physical positive interactions at Zoo G. Physical positive interactions were greatest between the matriarch and an unrelated infant. Non-physical interactions were highest between the matriarch and an unrelated infant and the infant and her mother. Betweenness scores were equal in both networks. .

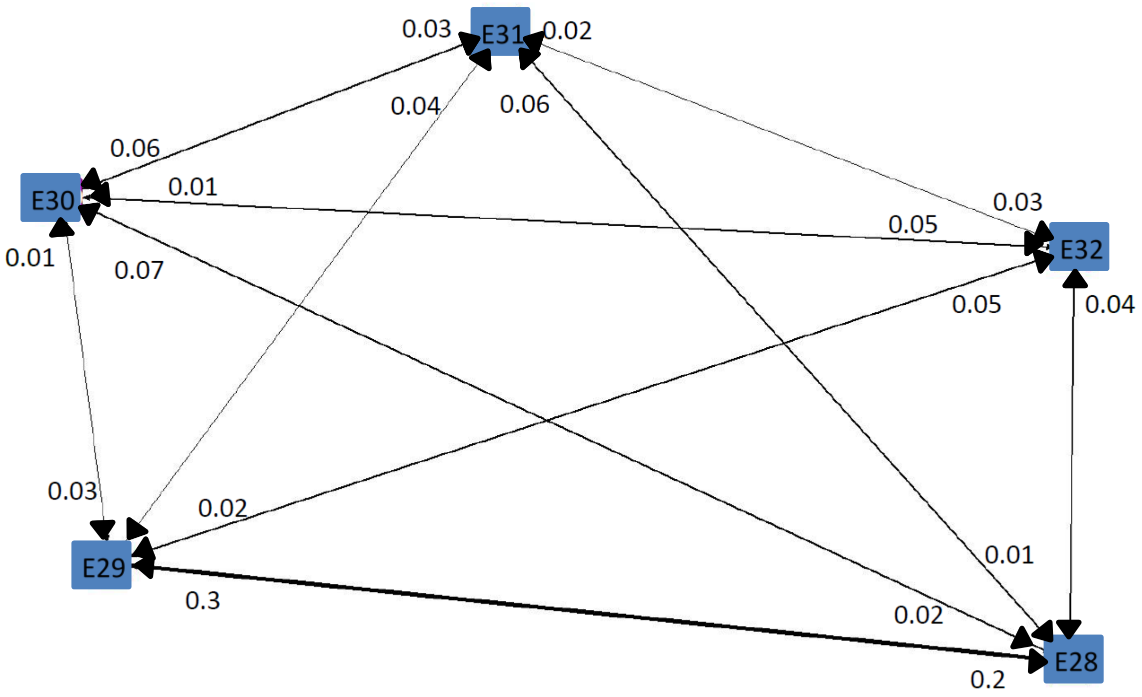

(a) Betweenness centrality scores: all individuals = 0

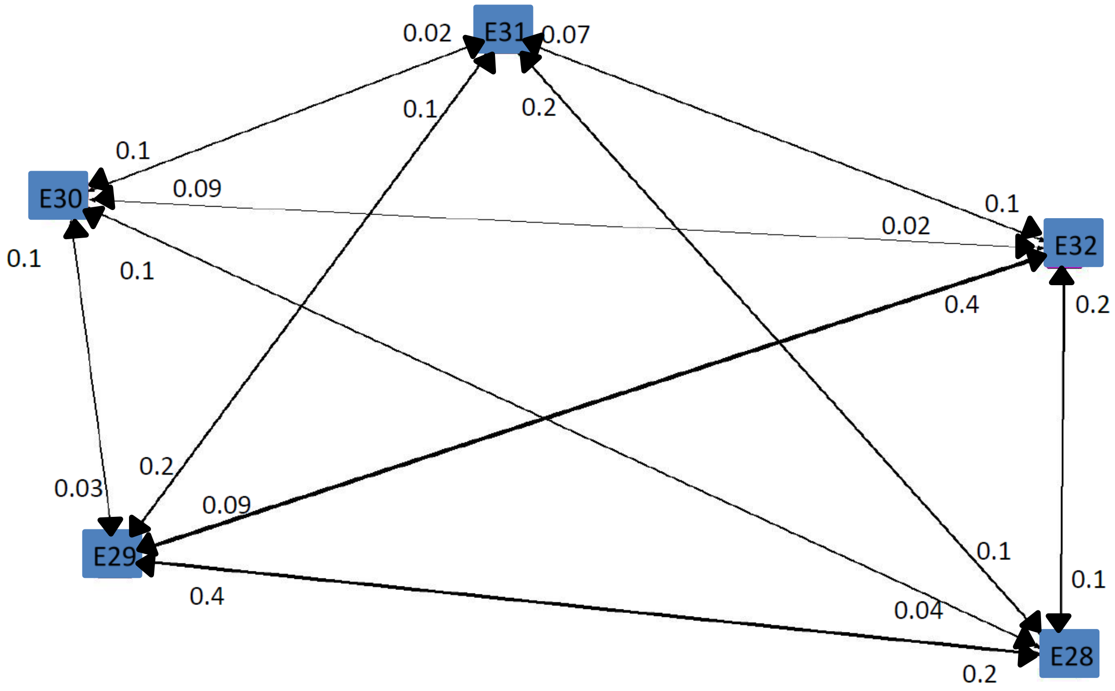

(b) Betweenness centrality scores: all individuals = 0

Figure S13. Sociograms depicting (a) physical negative interactions and (b) non-physical negative interactions at Zoo G. The matriarch gave most physical negative interactions; highest frequencies were between her and the mother of the infant. Non-physical negative interactions were highest from the mother of the infant to E32. Betweenness scores were equal in both networks.

References

[1] Wey, T.W.; Blumstein D.T.; Shen, W.; Jordan, F. Social network analysis of animal behaviour: a promising tool for the study of sociality. *Anim Behav* **2008**, 75, 1343-1352
